# ZNF8 Orchestrates with Smad3 to Promote Lung Metastasis by Recruiting SMYD3 in Breast Cancer

**DOI:** 10.1002/advs.202404904

**Published:** 2024-09-03

**Authors:** Wenwen Geng, Junhua An, Ke Dong, Hailu Zhang, Xiuyuan Zhang, Yuchen Liu, Rong Xu, Yifan Liu, Xiaofen Huang, Haiyun Song, Wei Yan, Aihua Sun, Fuchu He, Jian Wang, Haidong Gao, Chunyan Tian

**Affiliations:** ^1^ Department of Breast Surgery Qilu Hospital (Qingdao) Cheeloo College of Medicine Shandong University Qingdao Shandong 266000 China; ^2^ Laboratory of Oncology Qilu Hospital (Qingdao) Cheeloo College of Medicine Shandong University Qingdao Shandong 266000 China; ^3^ State Key Laboratory of Medical Proteomics Beijing Proteome Research Center National Center for Protein Sciences (Beijing) Beijing Institute of Lifeomics Beijing 102206 China; ^4^ College of Life Sciences Hebei University Baoding Hebei 071002 China; ^5^ Department of Pathology Qilu Hospital (Qingdao) Cheeloo College of Medicine Shandong University Qingdao Shandong 266000 China; ^6^ The First Medical Center of Chinese PLA General Hospital Beijing 100036 China; ^7^ Research Unit of Proteomics Dirven Cancer Precision Medicine Chinese Academy of Medical Sciences Beijing 102206 China

**Keywords:** breast cancer, lung metastasis, Smad3, TGF‐β pathway, ZNF8

## Abstract

Most deaths in breast cancer patients are attributed to metastasis, and lung metastasis is associated with a particularly poor prognosis; therefore it is imperative to identify potential target for intervention. The transforming growth factor‐β (TGF‐β) pathway plays a vital role in breast cancer metastasis, in which Smad3 is the key mediator and performs specific functions by binding with different cofactors. However, Smad3 cofactors involved in lung metastasis have not yet been identified. This study first establishes the interactome of Smad3 in breast cancer cells and identifies ZNF8 as a novel Smad3 cofactor. Furthermore, the results reveal that ZNF8 is closely associated with breast cancer lung metastasis prognosis, and specifically facilitates TGF‐β pathway‐mediated breast cancer lung metastasis by participating in multiple processes. Mechanistically, ZNF8 binds with Smad3 to enhance the H3K4me3 modification and promote the expression of lung metastasis signature genes by recruiting SMYD3. SMYD3 inhibition by BCI121 effectively prevents ZNF8‐mediated lung metastasis. Overall, the study identifies a novel cofactor of TGF‐β/Smad3 that promotes lung metastasis in breast cancer and introduces potential therapeutic strategies for the early management of breast cancer lung metastasis.

## Graphical abstract

Breast cancer lung metastasis is a complex process and has a poor prognosis, in which TGF‐β/Smad3 pathway plays a crucial role. However, Smad3 cofactors involved in lung metastasis have not yet been identified. This study identifies ZNF8 as a novel Smad3 cofactor that promotes lung metastasis via recruiting SMYD3, suggesting a potential therapeutic options.

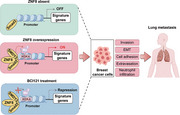

## 1 Introduction

Breast cancer is the most common cancer type and a leading cause of cancer mortality among women worldwide, and the majority of cancer‐related deaths are due to distant metastasis.^[^ ^1^ ^]^ The lungs, bones, brain, and liver are the principal organs susceptible to metastasis for breast cancer. However, among patients with metastatic breast cancer, lung metastasis is associated with a particularly poor prognosis, with a high mortality rate of 60–70%.^[^ ^2^ ^]^ Furthermore, lung metastasis is difficult to detect in the early stages, and often occurs within five years after the initial diagnosis of breast cancer.^[^ ^3^ ^]^ Therefore, early diagnosis of lung metastasis is highly important for the effective treatment of breast cancer patients.^[^ ^4^ ^]^

Studies have indicated that the development of lung metastasis in breast cancer may be associated with epithelial‐mesenchymal transition (EMT), breast cancer stem cells, the tumor microenvironment, and aberrant activation of signaling pathways in tumor cells.^[^ ^5^ ^]^ The transforming growth factor‐β(TGF‐β）signaling pathway has been closely linked to the progression of breast cancer.^[^ ^6^ ^]^ During the early stages, TGF‐β impedes cell cycle progression and stimulates cancer cell apoptosis, thereby exerting tumor‐suppressive effects. Nevertheless, during tumor progression, TGF‐β facilitates cancer cell metastasis through diverse mechanisms, such as promoting EMT, invasion, and extracellular matrix remodeling, modulating the tumor microenvironment and suppressing immune cell functionality.^[^ ^7^ ^]^ Multiple studies have revealed the critical role of TGF‐β pathway in breast cancer lung metastasis. Massague et al. reported that the TGF‐β pathway could promote lung metastasis by inducing ANGPTL4, which increased the permeability of lung capillaries and facilitated the transendothelial passage of breast cancer cells.^[^ ^8^ ^]^ Liu et al. reported that SIRT7 modulates TGF‐β signaling and suppresses of breast cancer metastasis by deacetylating and promoting Smad4 degradation.^[^ ^9^ ^]^ However, the exact mechanisms by which the TGF‐β pathway underlies lung metastasis in breast cancer remain incompletely understood, and there is still a lack of effective treatments targeting the TGF‐β pathway to suppress lung metastasis in breast cancer patients.

The TGF‐β signaling pathway relies on a series of Smad proteins including receptor‐regulated Smads (R‐Smads), common‐partner Smads (Co‐Smads), and I‐Smads inhibitory Smads (I‐Smads). As direct mediators of signal transduction, R‐Smads serve as key regulatory factors within the TGF‐β signaling pathway.^[^ ^10^ ^]^ After activation by TGF‐β1 and Activin, R‐Smads (Smad2 and Smad3) undergo phosphorylation, leading to their interaction with Co‐Smads and subsequent translocation into the cell nucleus. Moreover, due to their limited affinity for DNA through their own MH1 domain, R‐Smads need to collaborate with other DNA‐binding cofactors to achieve high affinity for DNA and selectivity for specific genes.^[^ ^11^ ^]^ Considering the critical role of TGF‐β in tumor metastasis, targeting specific cofactors would be an effective strategy for inhibiting breast cancer lung metastasis. However, the cofactors accounting for lung metastasis in breast cancer are rarely reported.

Compared with Smad2, Smad3 is the key R‐Smad involved in TGF‐β signaling and plays a more important role in breast cancer metastasis.^[^ ^12^ ^]^ However, there is a deficiency in systematic analysis and screening research pertaining to the interactomics of Smad3 cofactors in breast cancer cells. Thus, in this study, we established the interactome of Smad3 using immunoprecipitation mass spectrometry (IP‐MS) and characterized the cofactors in MDA‐MB‐231 breast cancer cells with lung metastatic potential. Through analyzing the interactions of Smad3, we revealed that the transcription factors of the zf‐C2H2 family might play a pivotal role in TGF‐β/Smad3‐mediated transcriptional regulation. Our further results revealed that the family member ZNF8, a novel Smad3 cofactor, was associated with the prognosis of lung metastasis in patients with breast cancer. Notably, ZNF8 promoted lung metastasis in breast cancer by facilitating multiple processes, including EMT, cell migration and invasion, endothelial adhesion, extravasation from vessels, and neutrophil infiltration in primary tumors and lungs. Mechanistically, ZNF8 interacted with Smad3 and coactivated the expression of lung metastasis signature genes by recruiting the methyltransferase SMYD3 to enrich H3K4me3 in gene promoters. In addition, blocking SMYD3 with BCI121 inhibited ZNF8‐mediated lung metastasis in breast cancer. Therefore, ZNF8 is a new Smad3 cofactor that involved in multiple steps of breast cancer lung metastasis, and targeting ZNF8 may be a potential strategy for preventing breast cancer lung metastasis.

## 2 Results

### 2.1 ZNF8 is a Novel Smad3‐Interacting Protein in Breast Cancer Cells

**Figure 1:**
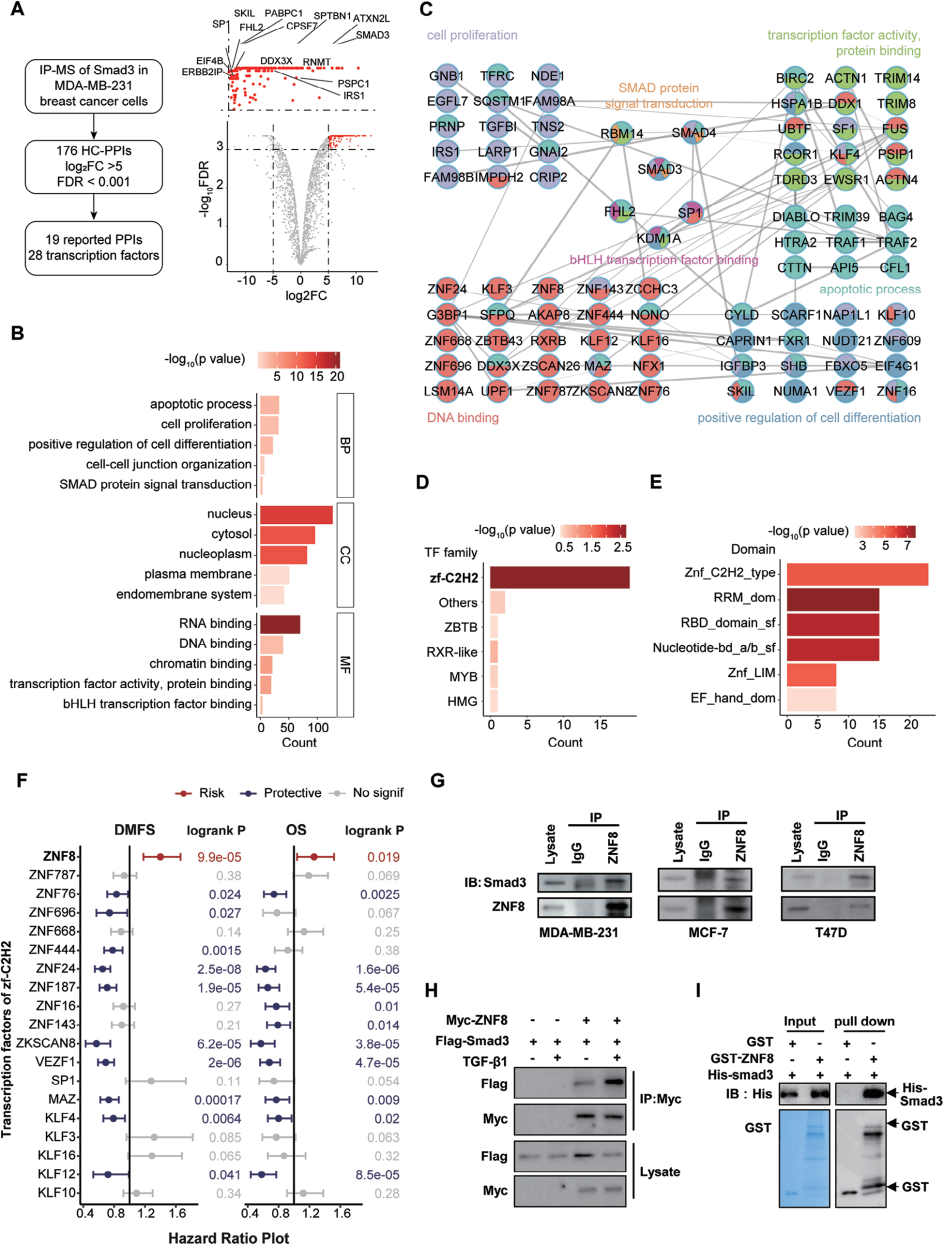
ZNF8 is a novel Smad3 interaction protein in breast cancer cells and associates with the prognosis in breast cancer patients. A) Schematic of the study of Smad3 interacting protein (left panel) in MDA‐MB‐231 breast cancer cells. Volcano plot represents the Smad interacting proteins, including those previously reported (right panel). B) Gene‐ontology analysis (BP, CC, and MF) of the 626 identified Smad3 interacting proteins in MDA‐MB‐231 cells. C) Interaction network of all known protein–protein interactions between the Smad3 partners identified in MDA‐MB‐231 cells. (One‐tailed t‐test, permutation‐based false discovery rate (FDR) < 0.05). Nodes describe: (1) the significance of the enrichment (size is proportional to the maximum −log P value); and (2) the function of the factors (color). D) The enrichment analysis for transcription factors of the Smad3 interacting proteins. E) The enrichment analysis for the binding domain of Smad3 interacting proteins. F) Online Kaplan‐Meier analysis (http://kmplot.com/) of the transcription factors of zf‐C2H2 that interacting with Smad3 on the overall survival (OS) and distant metastasis‐free survival (DMFS) prognosis of breast cancer. G) Co‐immunoprecipitation of endogenous Smad3 with anti ZNF8 antibodies in MDA‐MB‐231, MCF7, and T47 breast cancer cells. H) Co‐immunoprecipitation with anti‐Myc antibody in MDA‐MB‐231 cells co‐transfected with Myc‐ZNF8 and Flag‐Smad3 with TGF‐β1 treatment. I) GST pulldown with purified GST‐ZNF8 (upper) and Ni‐NTA pulldown with His‐Smad3 (lower) followed by immunoblotting with anti Smad3 and ZNF8 antibodies.

In this study, we identified 176 high‐confidence Smad3 interacting partners (Supplemental file‐Smad3 IP‐MS data), including 19 reported interactions (Reported protein interaction data were obtained from the BioGIRD database,^[^ ^13^ ^]^ and transcription factor family information was obtained from the AnimalTFDB database^[^ ^14^ ^]^) such as PSPC1, SKIL and EIF4B (**Figure** **1**A). Gene‐ontology molecular function (GO‐MF) enrichment analysis of these interacting proteins revealed statistical significance in the terms associated with transcription for RNA binding, DNA binding, chromatin binding, and transcription factor activity, protein binding, which are closely related to transcriptional regulation. GO‐cellular component (GO‐CC) enrichment analysis reveals that the proteins involved in nucleus, cytoplasm, and nucleoplasm were significantly enriched (Figure 1B). We also plotted the interaction network of Smad3, which revealed interaction complexes involved in apoptosis, cell proliferation, cell differentiation, cell‐cell junction organization, and Smad protein signal transduction (Figure 1C). As the canonical function of Smad3 is to control the expression of target genes by interacting with transcription factors,^[^ ^15^ ^]^ we focused exclusively on the transcriptional factors interacting with Smad3. The results revealed that zf‐C2H2 was the most significantly enriched TF (Figure 1D). In addition, domain enrichment analysis revealed that multiple types of domains were significantly enriched, with the znf‐C2H2 domain being the most significant (Figure 1E). Interestingly, compared with the other zf‐C2H2 transcription factors, ZNF8 was associated with a significantly worse breast cancer prognosis in terms of both overall survival (OS) and distant metastasis‐free survival (DMFS), which was assessed via online Kaplan‒Meier analysis (http://kmplot.com/) (Figure 1F),^[^ ^16^ ^]^ and was chosen as the target of subsequent studies. The interaction between ZNF8 and Smad3 was confirmed in MDA‐MB‐231, MCF‐7, and T47D breast cancer cells by co‐immunoprecipitation (Co‐IP), which also showed that the interaction was enhanced after TGF‐β pathway activation (Figure 1G,H). Moreover, GST pulldown experiments using recombinant His‐Smad3 and GST‐ZNF8 further verified this direct interaction (Figure 1I). Through the above research, we identified and verified that ZNF8 was a novel interacting factor of Smad3 in breast cancer cells, and preliminary analysis revealed that ZNF8 was a negative prognostic factor for breast cancer.

### 2.2 ZNF8 is Associated with Metastasis, Especially Lung Metastasis, Prognosis of Breast Cancer

**Figure 2:**
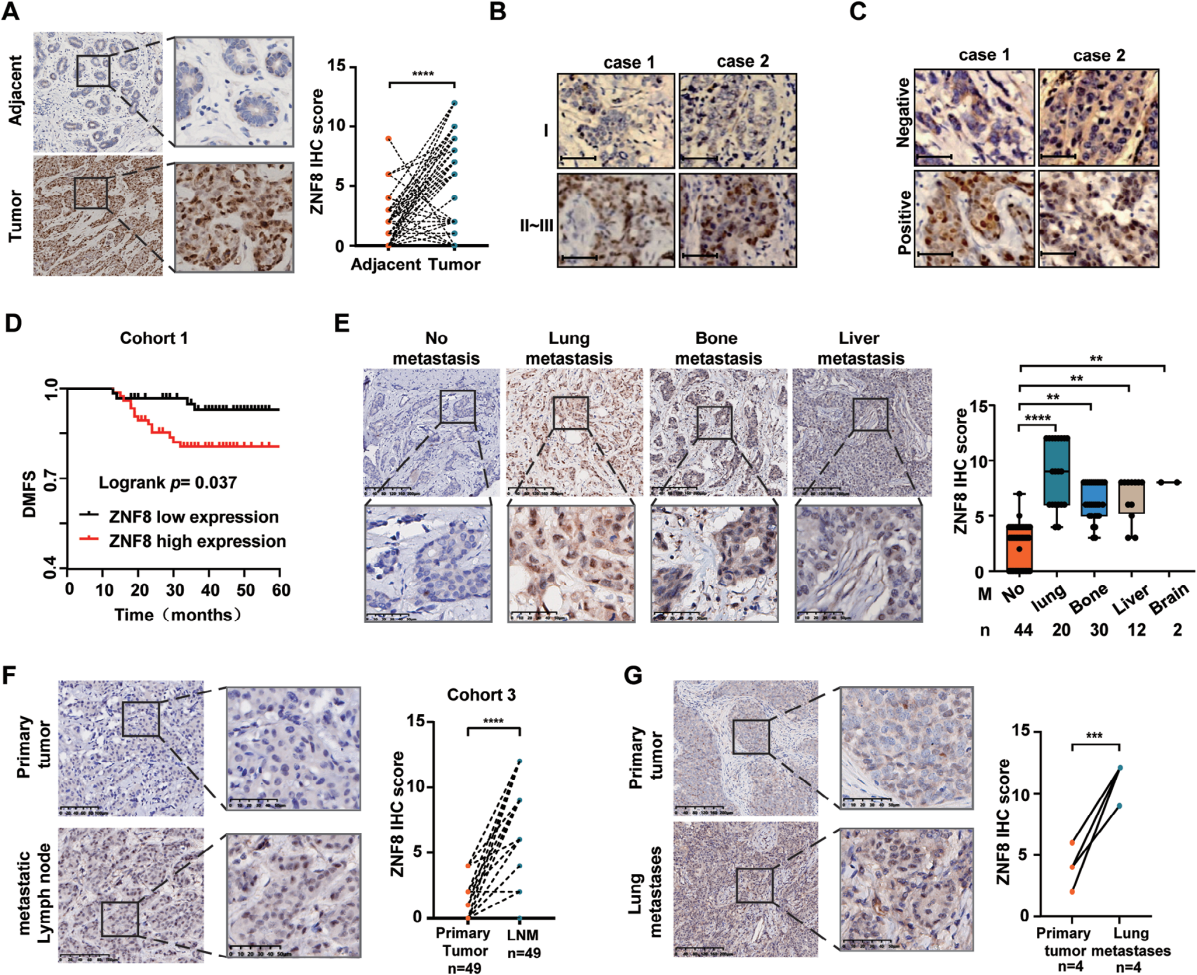
ZNF8 is associates with the lung metastasis prognosis in breast cancer patients. A) Representative immunohistochemical (IHC) staining and scoring of ZNF8 expression in breast tumors and paired adjacent tissues (Cohort 1, n = 152). B) Representative IHC staining and scoring of ZNF8 expression in breast cancer patients with different histological grades (Cohort 1, n = 152). C) Representative IHC staining and scoring of ZNF8 expression in breast cancer patients with different status of lymph nodes (right panel) (Cohort 1, n = 152). D) Kaplan‐Meier curves representing the probability of DMFS for the breast cancer patients with different expression levels of ZNF8 protein (Cohort 1, n = 152). E) Representative IHC staining and scoring of ZNF8 expression in breast cancer patients with different status of distant organs metastasis (Cohort 2). F) Representative IHC staining and scoring of ZNF8 expression in primary breast tumors and paired lymph node metastases (Cohort 3). G) Representative IHC staining and scoring of ZNF8 expression in primary breast tumors and paired lung metastases. For A and E‐G, significance was determined with the student unpaired t test. For D, significance was determined with Log–rank (Mantel–Cox) test. ns, not significant, *p* > 0.05; *, *p* < 0.05; ***, *p* < 0.001; ****, *p* < 0.0001. Error bars, ± SD. Scale bar = 200 µm (lower magnification) or 50 µm (higher magnification).

Considering the significant prognostic value of ZNF8 for breast cancer, we conducted a series of studies to further evaluate the clinical correlation of ZNF8 with multiple cohorts of breast cancer patients. Compared with that in paired adjacent normal tissues, ZNF8 expression was significantly elevated in tumors (**Figure** **2**A). The elevated expression of ZNF8 was significantly associated with a higher histological grade, lymph node metastasis, HER‐2 receptor positivity while no significant correlation was observed with tumor size, and molecular types of breast cancer both in our Cohort 1 and The Cancer Genome Atlas Program (TCGA) samples (Figure 2B,C; Table S1 and Figure S1, Supporting Information). Furthermore, Kaplan‐Meier survival analysis showed that, compared with patients with low ZNF8 expression, those with high ZNF8 expression had poorer DMFS (Figure 2D). To investigate the correlation between ZNF8 expression and different metastasis status of breast cancer, we studied another cohort comprising 44 patients without metastasis and 66 patients with metastatic breast cancer (Table S2, Supporting Information), and found a substantial increase in ZNF8 expression among breast cancer patients with lung, bone, liver and brain metastasis, especially those with lung metastasis (Figure 2E). This finding prompted us to determine the ZNF8 protein levels in metastatic tissues, which revealed that ZNF8 expression was significantly upregulated in both metastatic lymph nodes and metastatic lung tumors compared to that in paired primary tumors (Figure 2F,G). Therefore, these results suggested a strong association between ZNF8 expression and the prognosis of patients with metastatic breast cancer, especially those with lung metastasis.

### 2.3 ZNF8 Promotes Breast Cancer Lung Metastasis

**Figure 3:**
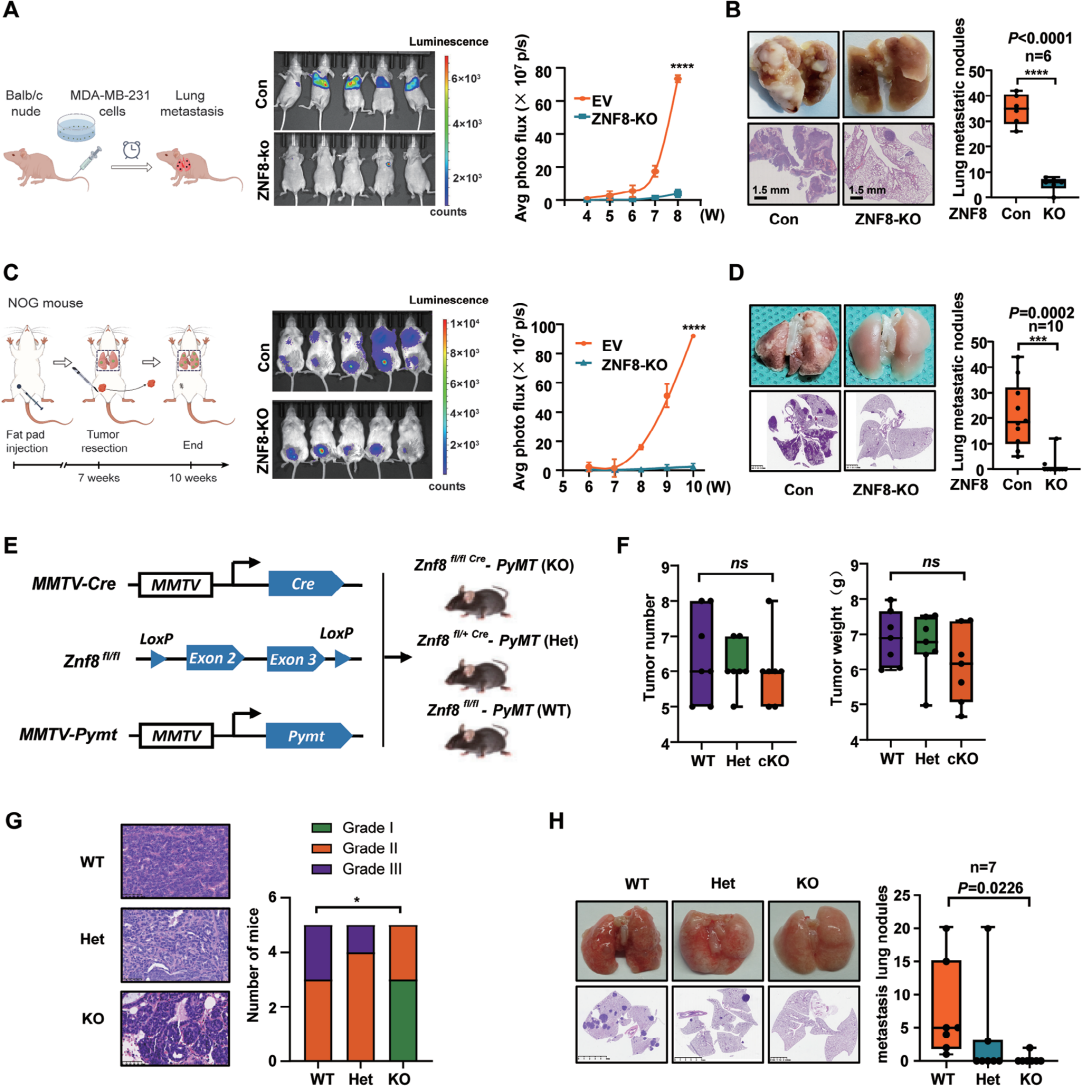
ZNF8 promotes breast cancer lung metastasis. A) Schematic illustration of the study of lung metastasis via tail‐vein injection of MDA‐MB‐231 breast cancer cells, (n = 10 mice per group) (left panel). Lung metastasis was analyzed by in vivo bioluminescence imaging; plot represents normalized photon flux from mouse lungs (right panel). (B) Representative images and quantification of lung metastatic nodules in lung tissues harvested at Week15, (n = 6 mice per group). C) Schematic illustration of the study of lung metastasis of orthotopic implantation by mammary fat pad injections of MDA‐MB‐231 breast cancer cells, (left panel). Lung metastasis was analyzed by in vivo bioluminescence imaging, plot represents normalized photon flux from mouse lungs (right panel), (n = 10 mice per group). D) Representative images and quantification of lung metastatic nodules in lung tissues harvested at Week10, (n = 10 mice per group). E) Schematic diagram of the strategy to obtain transgenic mice. F) Quantification of tumor number (number of tumors per mouse) and weight in KO, Het, and WT animals 23 weeks after birth (left panel). Quantification of tumor weight in KO, Het, and WT animals 23 weeks after birth (right panel) (n = 7 mice per group). One‐way ANOVA, not significant (ns). G) Representative Images and quantification of histological grades of mammary tumor in KO, Het, and WT animals 11 weeks after birth (n = 5 mice per group). *p = 0.020. H) Representative images and quantification of lung metastatic nodules in KO, Het, and WT animals 23 weeks after birth (n = 7 mice per group). *p = 0.0226. For A–D, G, H, significance was determined with the student unpaired t test. ns, *p* > 0.05; *, *p* < 0.05; ***, *p* < 0.001; ****, *p* < 0.0001. For F and H, significance was determined with One‐way ANOVA. Error bars, ± SD.

To further confirm the biological roles of ZNF8 in breast cancer, we generated ZNF8 knockout MDA‐MB‐231 and Hs578T cells and ZNF8‐overexpressing ZR‐75‐1 and MCF‐7 cells (Figure S2A, Supporting Information). Using tail vein metastasis models, we observed that lung metastasis was notably inhibited in the ZNF8 knockout group (**Figure** **3**A,B), and the ZNF8 knockout group had a better prognosis in terms of lung metastasis‐free survival (LMFS) (Figure S2B, Supporting Information). Results from an orthotopic mouse xenograft model further confirmed that ZNF8 knockout significantly suppressed lung metastasis (Figure 3C,D), and Kaplan‒Meier curve analysis revealed better LMFS in the ZNF8 knockout group (Figure S2C, Supporting Information). However, the impact of ZNF8 on the growth of orthotopic tumors was nonsignificant, as the tumor cells isolated from orthotopic and lung metastatic tumors from the ZNF8 knockout group and the control group displayed the same proliferative capacity (Figure S2D,E, Supporting Information). The above results indicated that ZNF8 promoted breast cancer cell lung metastasis but did not affect primary tumor growth.

To further investigate the ability of ZNF8 to promote breast cancer metastasis, we generated conditional mammary epithelium cell‐specific ZNF8 knockout mice (MMTV‐Cre; Znf8^fl/fl^). Then, these mice were crossed with MMTV‐PyMT transgenic mice, a classic spontaneous mammary tumor model, to obtain Znf8^fl/fl^; MMTV‐ PyMT (WT), Znf8 ^fl/fl^; MMTV‐Cre; MMTV‐PyMT (KO) and heterozygous knockout mice (Znf8^fl/+^; MMTV‐Cre; MMTV‐PyMT (Het)) (Figure 3E). PyMT‐driven mammary tumorigenesis and lung metastasis were examined in virgin females in the presence or absence of endogenous ZNF8. There was no significant difference in primary tumor number or burden between ZNF8 homozygous knockout mice and WT and Het mice (Figure 3F). In addition, expression of Ki‐67 was also evaluated in the tumor tissues, and the results revealed there was no significant difference in the Ki‐67 positive cells between the WT and ZNF8‐KO mice (Figure S2F, Supporting Information). However, histological analysis revealed that the tumor grade of ZNF8 knockout mice was significantly lower than that of the mice in the other two groups (Figure 3G). Notably, spontaneous lung metastasis was inhibited in ZNF8 homozygous knockout mice, as evidenced by a significant reduction in both the rate of lung metastasis and the number of metastatic nodules (Figure 3H; Figure S2G, Supporting Information). We also isolated the tumor cells from the lung metastasis lesion of WT and KO mice and conducted Transwell analysis, and the results showed the migration and invasion abilities of breast cancer cells were markedly decreased in ZNF8 knockout tumor cells (Figure S2H, Supporting Information). Therefore, these results confirmed that ZNF8 played an important role in breast cancer cell lung metastasis but had no significant effect on tumor growth in mice.

### 2.4 ZNF8 is Involved in Multiple Processes of Lung Metastatic Cascades

**Figure 4:**
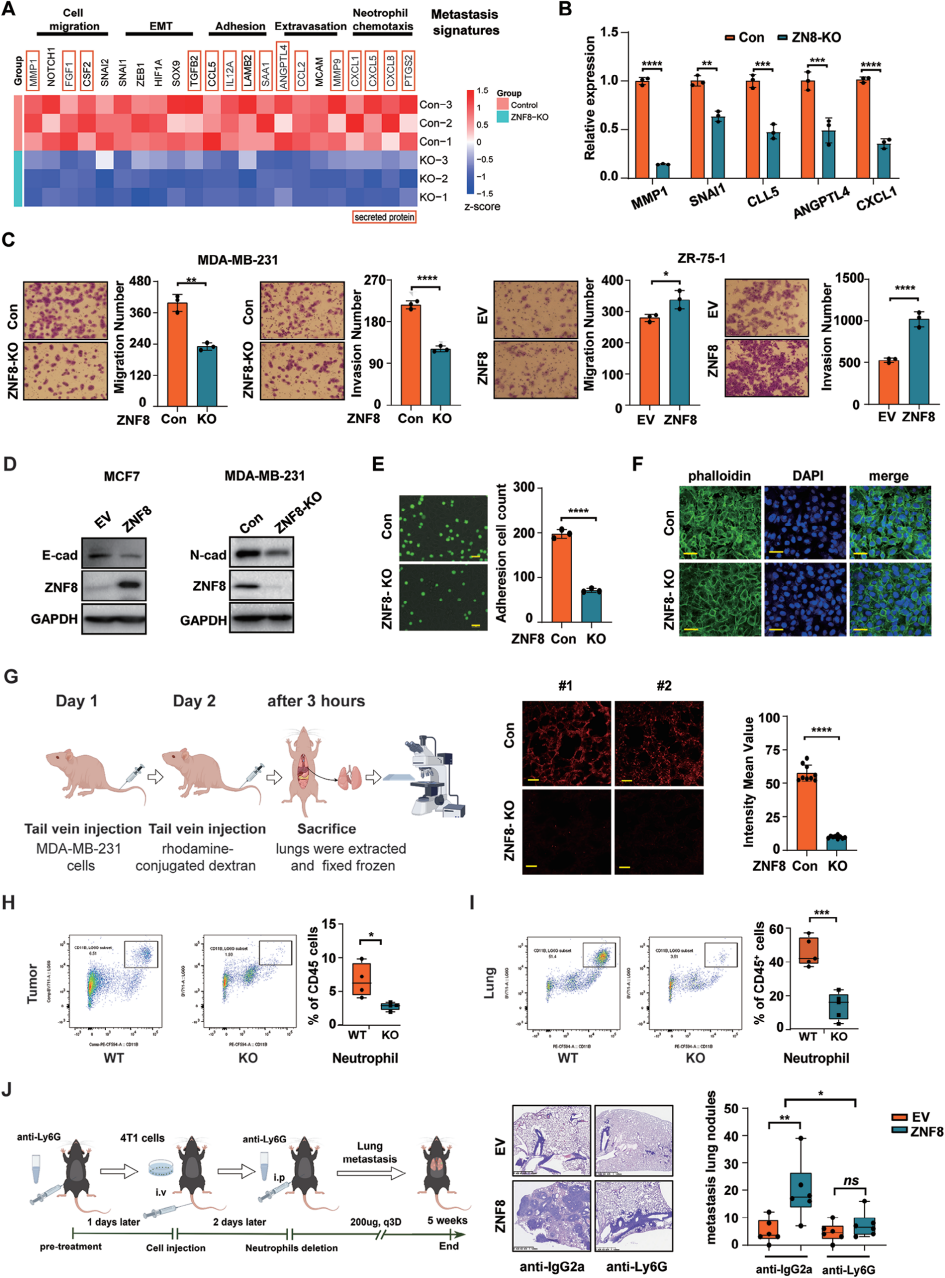
ZNF8 is involved in the multiple processes in the metastatic cascades. A) RNA‐seq heatmap of the metastasis signature genes associated with cell migration, EMT, adhesion, extravasation, and neutrophil chemotaxis in control and ZNF8 knockout MDA‐MB‐231cells (n ≥ 3). B) RT–qPCR validation the RNA‐seq data of signature genes, MMP1, SNAI1, CCL5, ANGPTLE4, and CXCL1 in control and ZNF8 knockout MDA‐MB‐231 cells (n ≥ 3). C) Representative images and quantification of Transwell assay for migration and invasion in MDA‐MB‐231 cells (control versus ZNF8 knockout) (left panel), and in ZR‐75‐1 cells (empty vector versus ZNF8 overexpression) (right panel) (n ≥ 3). D) Representative Western blots for E‐cadherin, ZNF8 and the loading control GAPDH in ZNF8 overexpression ZR‐75‐1 and MCF7 cells, and for N‐cadherin, ZNF8 and the loading control GAPDH in ZNF8 knockout MDA‐MB‐231 cells. E) Representative images and quantification of cell adhesion assay in control and ZNF8 knockout MDA‐MB‐231 cells (n ≥ 3). Cell number was counted in six randomly captured pictures. Scale bar, 50 µm. F) Representative confocal images of phalloidin (green) and DAPI (blue) in HUVEC monolayers treated with conditioned media from control and ZNF8 knockout MDA‐MB‐231 cells (n ≥ 3). bar = 50 µm. G) Schematic diagram of the lung permeability assays and representative confocal images of the accumulation of rhodamine‐dextran in the lung parenchyma (n ≥ 3). bar = 50 µm. H, I) Representative diagram and quantification of neutrophil infiltration in tumors (I) and lungs J) from transgenic mice 20week from birth (n = 5). (J) The study of neutrophil depleted on the lung metastasis promoted by ZNF8 via tail‐vein injection of 4T1 cells in C57BL/6J mice with the anti‐Ly6G clearance antibody (left panel). Representative HE images and quantification of lung metastatic nodules (left panel). (n = 6 mice/group), bar = 1.25 mm. For B, C, E, G, H, I, and J, data represent mean ± SD, and significance was determined with the student unpaired t test. *ns*, *p* > 0.05; *, *p* < 0.05; **, *p* < 0.01; ***, *p* < 0.001; ****, *p* < 0.0001.

To further investigate the mechanism by which ZNF8 promotes lung metastasis, we performed RNA‐Seq analysis on ZNF8 knockout and control MDA‐MB‐231 cells. GO term analysis of the genes downregulated by ZNF8 knockout revealed enrichment of genes involved in EMT, regulation of binding, regulation of extracellular matrix assembly, and positive regulation of epithelial cell migration (Figure S3A, Supporting Information) and enrichment of genes associated with cell migration, EMT, cell adhesion, extravasation and neutrophil chemotaxis (**Figure** **4**A). Among them, MMP1, SNAI1, CCL5, ANGPTL4, and CXCL1 are well known as the lung metastasis signature genes with close association with the above processes.^[^ ^17^ ^]^ RT‒qPCR was performed to confirm the effect of ZNF8 on the expression of these genes in MDA‐MB‐231 and MCF‐7 cells (Figure 4B; Figure S3B, Supporting Information), and the Chromatin immunoprecipitation‐qPCR (ChIP‐qPCR) assays showed ZNF8 can bind to the promoter of target genes (Figure S3C, Supporting Information) in MDA‐MB‐231 cells. Consistent with the function of the lung metastasis signature genes, ZNF8 knockout significantly hindered migration and invasion, whereas ZNF8 overexpression notably enhanced these abilities in ZR‐75‐1 and Hs578T cells (Figure 4C; Figure S3D, Supporting Information). Considering that most of the prometastatic genes regulated by ZNF8 encode secreted proteins, we examined the effects of conditioned media obtained from cultured breast cancer cells with different ZNF8 expression levels. The conditioned media derived from ZNF8‐overexpressing and ZNF8 knockout cells suppressed the invasion abilities of breast cancer cells, indicating that ZNF8 could promote cancer cell invasion through cell‐autonomous and non‐cell‐autonomous mechanisms simultaneously (Figure S3E, Supporting Information). Subsequently, we investigated the effect of ZNF8 on EMT by examining the expression of EMT markers. ZNF8 overexpression downregulated the expression of E‐cadherin, and ZNF8 knockout or knockdown led to the inhibition of N‐cadherin expression (Figure 4D; Figure S3F, G, Supporting Information). These results indicated that ZNF8 could promote EMT in breast cancer cells.

Endothelial cell attachment and transendothelial migration are the key steps for tumor cell metastasis to the lung, and we next investigated the effects of ZNF8 on these processes. ZNF8 knockout severely reduced the adhesion of MDA‐MB‐231 cells to human umbilical vein endothelial cells (HUVEC) monolayers (Figure 4E). Concomitantly, in vitro vascular permeability experiments revealed that ZNF8 knockout significantly inhibited the ability of the tumor cells to disrupt the integrity of the HUVEC monolayer (Figure 4F). In vivo, we observed that the rhodamine signal in the lung tissue of mice injected with ZNF8 knockout cells was significantly lower than that in the lung tissue of control mice (Figure 4G), indicating that ZNF8 plays a substantial role in enhancing the extravasation capability of breast cancer cells. Moreover, the cell proliferation and colony formation assay results indicated that ZNF8 did not significantly affect the proliferation or colony formation abilities of breast cancer cells (Figure S3H, I, Supporting Information), which was consistent with the in vivo results.

Mass spectrometry‐based proteomic analyses of primary tumors from ZNF8‐KO (Znf8^fl/fl^;MMTV‐Cre;MMTV‐PyMT) and ZNF8‐WT (Znf8^fl/fl^;MMTV‐PyMT) mice further confirmed the prometastatic effect of ZNF8. Notably, Gene Set Enrichment Analysis (GSEA) of the tumor proteome revealed that ZNF8 expression level was positively correlated with chemotaxis in primary tumors (Figure S4A, Supporting Information). Therefore, we analyzed the changes in immune cell fractions by flow cytometry and Immunohistochemistry and found that neutrophil infiltration in the tumors of the knockout mice was significantly inhibited (Figure 4H; Figure S4B, Supporting Information), while no obvious difference was observed for other tumor‐infiltrating immune cells (Figure S4C, Supporting Information). Interestingly, in lung tissues collected from mice during the early stage of lung metastasis (20 weeks), ZNF8 promoted neutrophil infiltration more significantly than it did in the primary tumor (Figure 4I; Figure S4D, E, Supporting Information). GSEA of the lung proteome further confirmed that ZNF8 expression was positively correlated with neutrophil‐mediated immunity, neutrophil chemotaxis, neutrophil degranulation, and neutrophil chemotaxis, which promote metastasis (Figure S4F, Supporting Information). Heatmap analysis revealed that the expression of proteins involved in the above processes was significantly suppressed in ZNF8 knockout mice (Figure S4G, Supporting Information). Therefore, these results suggested that ZNF8‐facilitated breast cancer metastasis was closely associated with neutrophil infiltration. To further investigate the effect of neutrophil infiltration in lung metastasis in vivo, we employed a lung metastasis model by tail injection with empty vector and ZNF8 overexpression 4T1 cells in C57BL/6J mice. The results showed that the promoting effect of ZNF8 for the lung metastasis was significantly inhibited when neutrophils were specifically depleted in the mice with the anti‐Ly6G clearance antibody (Figures 4J). Overall, ZNF8 promoted lung metastasis of breast cancer by influencing multiple processes.

### 2.5 ZNF8 Regulates Lung Metastatic Signatures through the TGF‐β Pathway in a Smad3‐Dependent Manner

**Figure 5:**
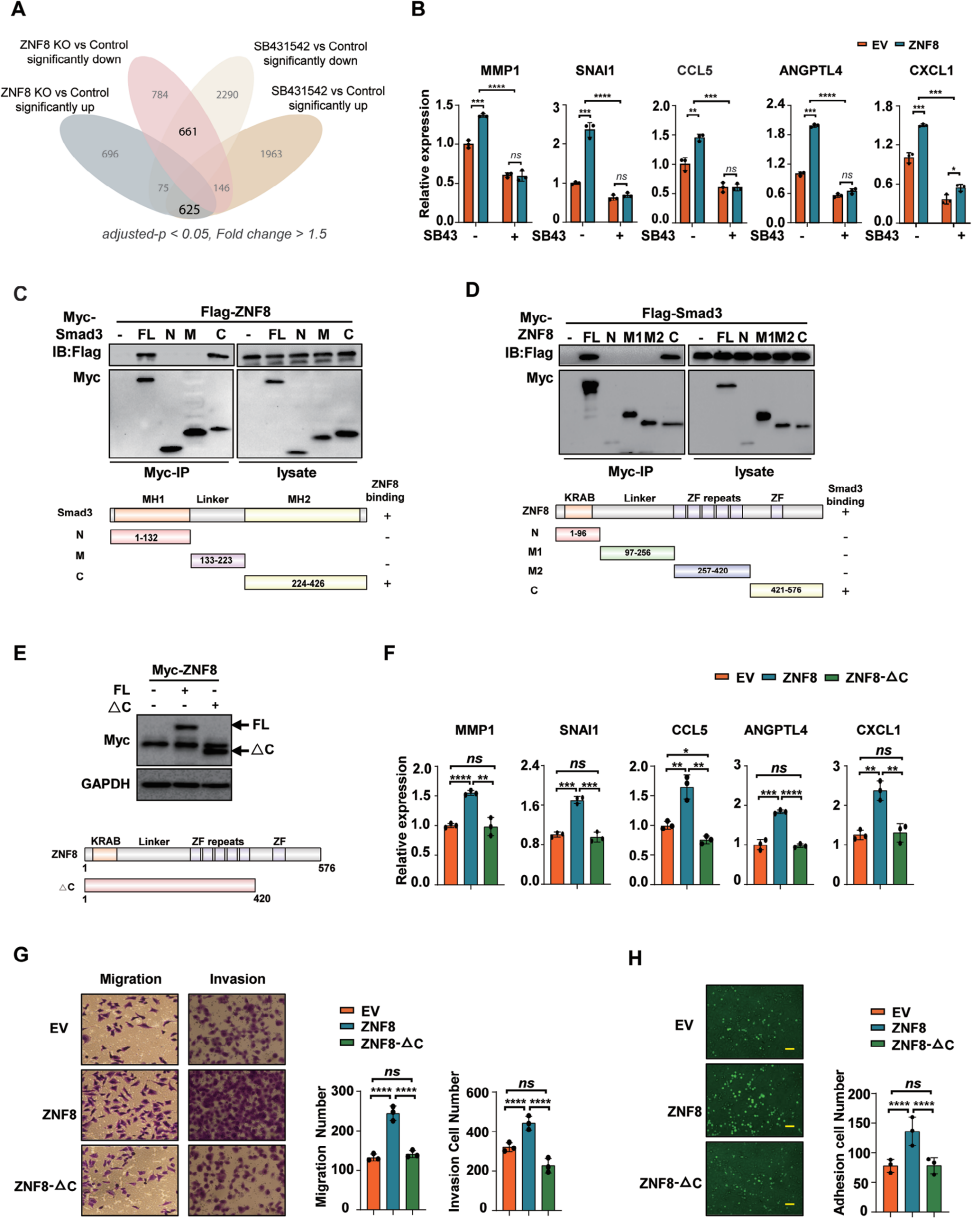
ZNF8 regulates the lung metastatic signatures through TGF‐β pathway in a Smad3‐dependent manner. A) Venn diagram of the overlap genes that regulated by ZNF8 knockout as well as TGF‐β pathway inhibitor SB431542 treatment in MDA‐MB‐231 breast cancer cells (*p‐*adj <0.05, fold change >1.5). (B) RT–qPCR analysis of signature genes MMP1, SNAI1, CCL5, ANGPTL4, and CXCL1 in ZNF8 overexpression ZR‐75‐1 cells with SB431542 treatment (n ≥ 3). C) Map of the region of smad3 that interacts with ZNF8. Co‐immunoprecipitation with anti‐Myc antibody in HEK293T cells co‐transfected with Flag‐ZNF8, Myc‐ZNF8 and Myc‐tagged deletion mutants of Smad3. D) Map of the region of ZNF8 that interacted with Smad3. Co‐immunoprecipitation with anti‐Myc antibody in HEK293T cells co‐transfected with Flag‐Smad3, Myc‐ZNF8 and Myc‐tagged deletion mutants of ZNF8. E) Representative Western blots for full‐length ZNF8, △C mutants, and the loading control GAPDH in ZNF8 knockout MDA‐MB‐231 cells transduced with Myc‐tagged full‐length ZNF8 or △C mutants. F) RT–qPCR analysis of signature genes in ZNF8 knockout MDA‐MB‐231 cells transduced with Myc‐tagged full‐length ZNF8 or △C mutants (n ≥ 3). G) Representative images and quantification of Transwell assay for migration and invasion in ZNF8 knockout MDA‐MB‐231 cells transduced with Myc‐tagged full‐length ZNF8 or △C mutants (n ≥ 3). H) Representative images and quantification of cell adhesion assay in ZNF8 knockout MDA‐MB‐231 cells transduced with Myc‐tagged full‐length ZNF8 or △C mutants. Cell number was counted in six randomly captured pictures (n ≥ 3). Scale bar, 50 µm. For B, and F‐H, data represent mean ± SD, and significance was determined with the student unpaired t test and Two‐way ANOVA. *ns*, *p* > 0.05; *, *p* < 0.05; **, *p* < 0.01; ***, *p* < 0.001; ****, *p* < 0.0001.

Although ZNF8 is a Smad3‐interacting protein, whether its prometastatic function is associated with the TGF‐β/Smad signaling pathway remains unclear. Thus, we conducted an RNA‐Seq analysis of the transcriptional profiles of ZNF8 knockout MDA‐MB‐231 cells and MDA‐MB‐231 cells treated with SB431542, a TGF‐β pathway inhibitor. Interestingly, a substantial proportion of the ZNF8 target genes overlapped with TGF‐β pathway genes (**Figure** **5**A). GO analysis of these overlapping genes revealed enrichment of genes associated with EMT, cell adhesion, and chemokine signaling (Figure S5A, Supporting Information). The ability of ZNF8 to promote the expression of the lung metastasis signature genes MMP1, SNAI1, CCL5, ANGPTL4, and CXCL1 was notably diminished by SB431542 treatment (Figure 5B). TGF‐β1 treatment significantly amplified ZNF8‐mediated promoting of cell migration, whereas ZNF8‐mediated breast cancer cell migration and invasion were significantly suppressed by SB431542 treatment (Figure S5B, C, Supporting Information). In addition, the inhibitory effect of ZNF8 on the expression of the EMT marker E‐cadherin was attenuated upon SB431542 treatment (Figure S5D, Supporting Information). Furthermore, the ability of HUVEC adhesion and lung metastasis promoted by ZNF8 overexpression were also inhibited by SB431542 treatment (Figure S5E, F, Supporting Information). These findings suggested that ZNF8 facilitated lung metastatic signatures via the TGF‐β pathway in breast cancer cells.

As described in the previous sections, ZNF8 is a novel Smad3 interaction protein and was associated with the lung metastasis of breast cancer. Therefore, to further investigate whether ZNF8 functionality relies on interactions with Smad3, we mapped the domains required for interactions between ZNF8 and Smad3. The results indicated that the C‐terminal domain of ZNF8 and the MH2 domain of Smad3 are responsible for their interaction (Figure 5C,D). Additionally, we performed functional rescue experiments by transfecting full‐length ZNF8 and its C‐terminal deletion mutant into ZNF8 knockout breast cancer cells (Figure 5E). The C‐terminal deletion mutant ZNF8 failed to restore signature gene expression or metastatic features related to cell migration, invasion, and adhesion in ZNF8 knockout MDA‐MB‐231 cells (Figure 5F‐H). In addition, our results showed that the promoting effects of ZNF8 on the expression of signature genes, the cell migration and invasion were significantly suppressed by Smad3 knockdown using siRNA.in MDA‐MB‐231 cells (Figure S5G, H, Supporting Information).These findings provide compelling evidence that ZNF8 promotes metastasis in a Smad3‐dependent manner.

### 2.6 ZNF8 is Indispensable for TGF‐β Signaling Pathway‐Mediated Lung Metastasis in Breast Cancer Cells

**Figure 6:**
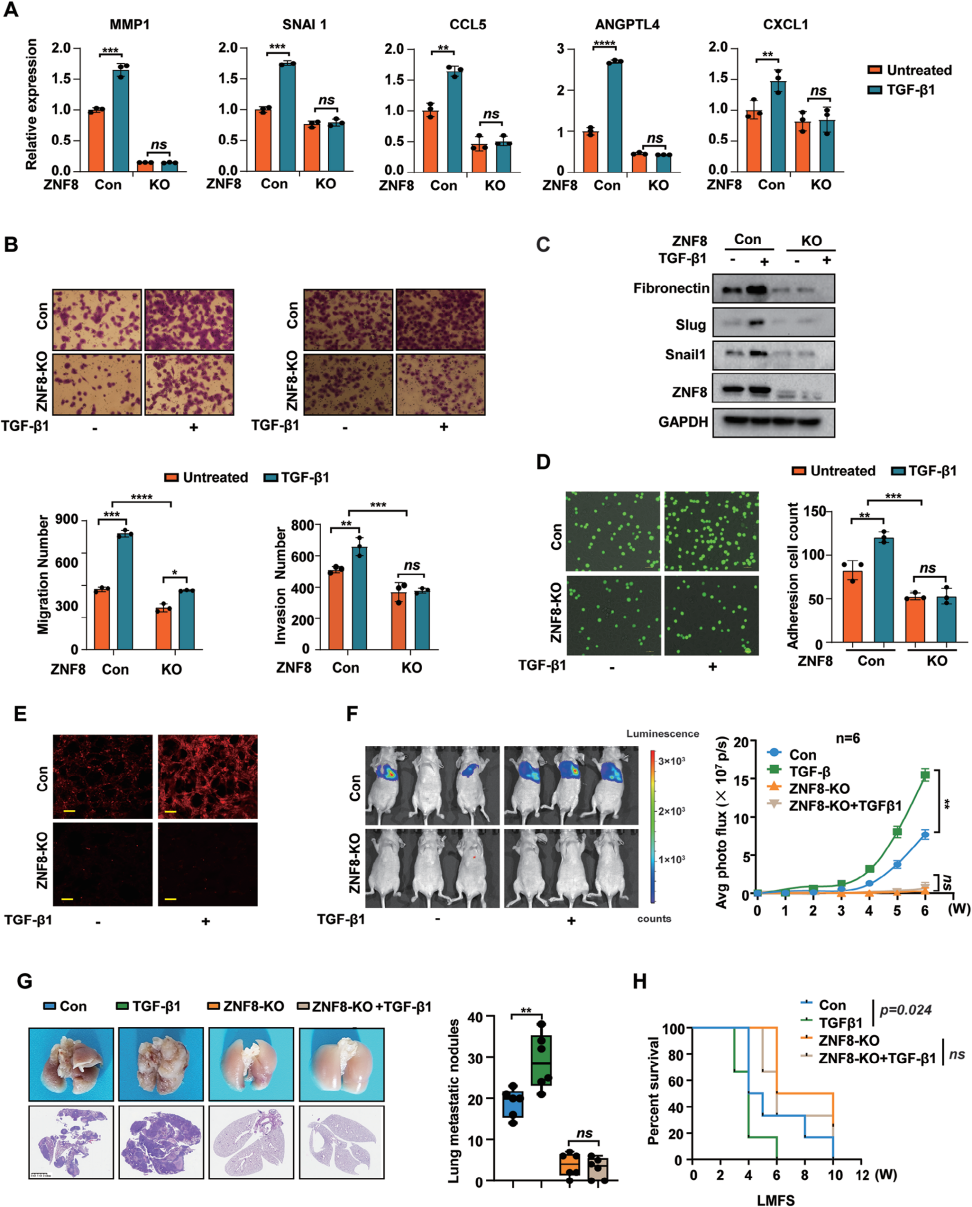
ZNF8 is indispensable for the TGF‐β signaling pathway‐mediated metastasis in breast cancer cells. A) RT–qPCR analysis of signature genes in control and ZNF8 knockout MDA‐MB‐231 cells with TGF‐β1 treatment (n ≥ 3). B) Representative images and quantification of Transwell assay for migration and invasion in control and ZNF8 knockout MDA‐MB‐231 cells with TGF‐β1 treatment. C) Representative Western blots for Fibronectin, Slug, Snail1, ZNF8, and the loading control GAPDH in control and ZNF8 knockout MDA‐MB‐231 cells with TGF‐β1 treatment. D) Representative images and quantification of cell adhesion assay in control and ZNF8 knockout MDA‐MB‐231 cells with TGF‐β1 treatment. Cell number was counted in six randomly captured pictures. E) Representative confocal images of the accumulation of rhodamine‐dextran in the lung parenchyma in control and ZNF8 knockout MDA‐MB‐231 cells with TGF‐β1 treatment. F) The study of lung metastasis via tail‐vein injection of MDA‐MB‐231 breast cancer cells pretreated with TGF‐β1 (left panel). Lung metastasis was analyzed by in vivo bioluminescence imaging; plot represents normalized photon flux from mouse lungs (right panel). (n = 6 mice per group). G) Representative images and quantification of lung metastatic nodules in lung tissues harvested at Week15, (n = 6 mice per group). H) Kaplan–Meier survival curves of nude mice treated with tail‐vein injection of control and ZNF8 knockout MDA‐MB‐231 cells with TGF‐β1 pretreatment. For A, B, D, F, and G, data represent mean ± SD, and significance was determined with the Student's t test, For H, significance was determined with Log–rank (Mantel–Cox) test. ns, *p* > 0.05; *, *p* < 0.05; **, *p* < 0.01; ***, *p* < 0.001; ****, *p* < 0.0001. Scale bar, 50 µm.

The above findings revealed that ZNF8 promoted breast cancer metastasis through interactions with Smad3, but its importance in the TGF‐β pathway remains unclear. Therefore, we investigated the prometastatic function of the TGF‐β pathway in ZNF8 knockout cells. The ChIP‐qPCR results revealed that ZNF8 overexpression could enhance the Smad3 occupancy at signature genes promoter (Figure S6A, Supporting Information). Furthermore, knocking out ZNF8 significantly inhibited the TGF‐β1‐induced transcription of lung metastasis signature genes and the migratory and invasive properties of breast cancer cells (**Figure** **6**A,B). Additionally, ZNF8 knockout inhibited the TGF‐β1‐induced expression of Fibronectin, Slug, Snail1, and Vimentin (Figure 6C; Figure S6B, Supporting Information). Moreover, ZNF8 knockout also hindered the TGF‐β pathway‐promoted adherence of breast cancer cells to HUVECs (Figure 6D). We next examined the ability of TGF‐β1 to promote vascular cell permeability in control and ZNF8 knockout cells. TGF‐β1 notably enhanced vascular endothelial cell permeability in vitro and in vivo, and this effect was significantly impaired or entirely inhibited by ZNF8 knockout (Figure 6E; Figure S6C, Supporting Information).

Furthermore, in the model of metastasis induced by tail vein injection in nude mice, ZNF8 knockout significantly suppressed the increase in lung metastasis induced by TGF‐β1 (Figure 6F,G). Survival analysis of LMSF also revealed that mice in the TGF‐β1 treatment group were more prone to lung metastasis by breast cancer cells, and ZNF8 knockout almost completely suppressed this effect (Figure 6H). Additionally, we treated breast cancer cells with the TGF‐β pathway inhibitor SB431542. Knocking out ZNF8 significantly attenuated the inhibitory effect of SB431542 on Vimentin expression, cell migration, and invasion in MDA‐MB‐231 and Hs578T cells (Figure S6D, E, Supporting Information). In conclusion, our results demonstrated the indispensable role of ZNF8 in TGF‐β‐induced EMT and metastasis in breast cancer.

### 2.7 ZNF8 Recruits SMYD3 to Smad3 and Promotes the Transcriptional Activation of Lung Metastasis Signature Genes Within the TGF‐β Pathway

We next sought to investigate the molecular mechanism by which ZNF8 regulates the TGF‐β pathway to promote breast cancer metastasis. First, we examined the phosphorylation and nuclear translocation of Smad2/3, which are crucial steps in TGF‐β pathway activation. However, ZNF8 did not induce substantial changes in the total or phosphorylated levels of Smad2/3 in MDA‐MB‐231 cells (Figure S7A, Supporting Information). In parallel, ZNF8 overexpression or knockout did not significantly alter the localization of R‐Smad2/3 to the nucleus in MDA‐MB‐231 cells treated with or without TGF‐β1 (Figure S7B, C, Supporting Information). Thus, these findings indicated that ZNF8 might regulate the TGF‐β signaling pathway through other mechanisms.

**Figure 7:**
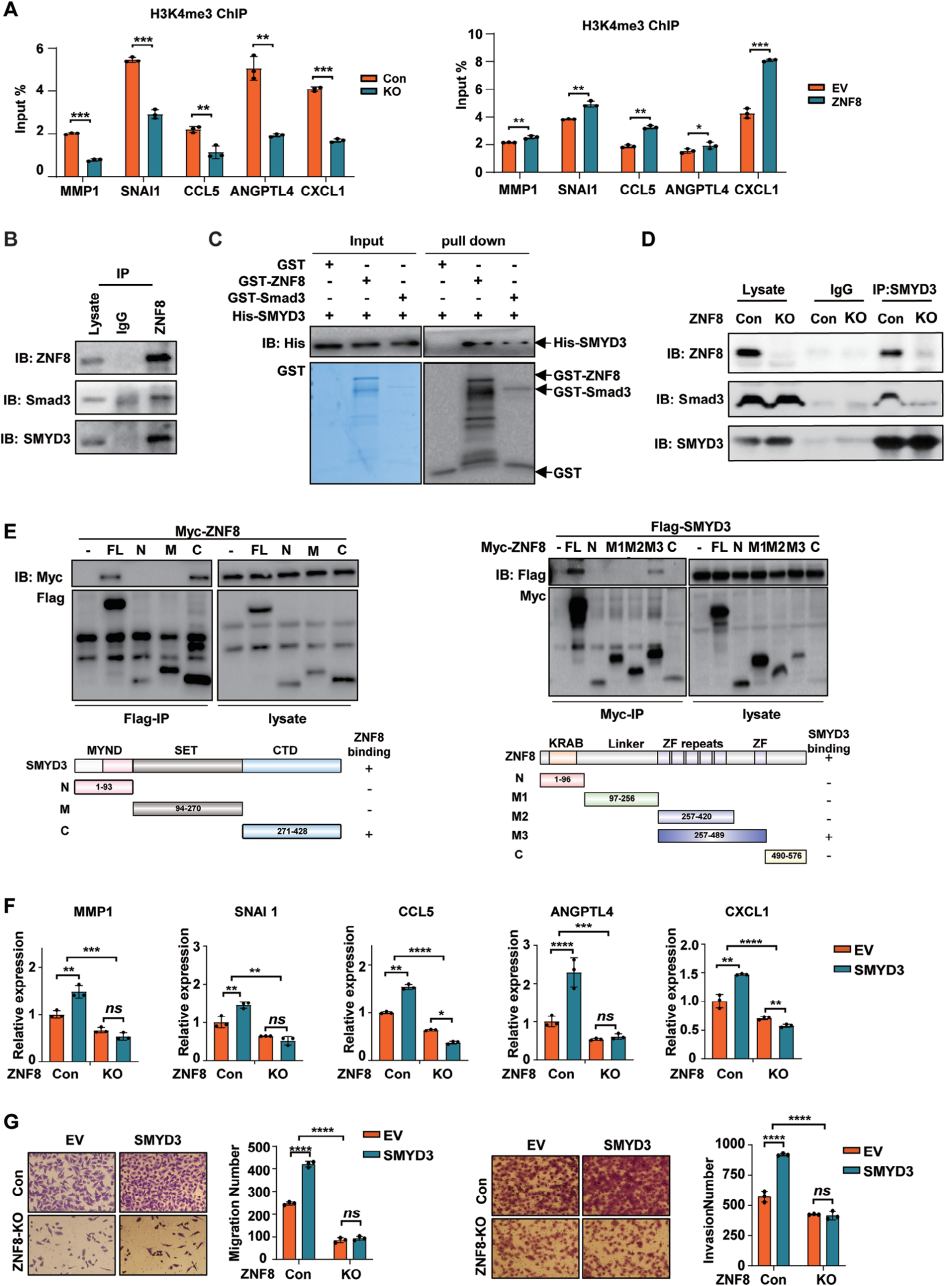
ZNF8 recruits SMYD3 to Smad3 and promotes the transcription activation of the lung metastasis signature genes within TGF‐β pathway. A) ChIP‐qPCR for H3K4me3 occupancy at signature genes promoter in ZNF8 knockout and overexpression MDA‐MB‐231 cells as quantified by % of Input (n ≥ 3). B) Co‐immunoprecipitation of endogenous ZNF8 with Smad3 and SMYD3 using ZNF8 antibodies in MDA‐MB‐231 breast cancer cells. C) GST pulldown with purified GST‐ZNF8 and His‐Smad3 followed by immunoblotting with anti Smad3 and ZNF8 antibodies. D) Co‐immunoprecipitation of endogenous SMYD3 with Smad3 using SMYD3 antibodies in Control and ZNF8 knockout MDA‐MB‐231 breast cancer cells. E) Map of the region of SMYD3 and ZNF8 that interacted with each other. Co‐immunoprecipitation with anti‐Flag antibody in HEK293T cells co‐transfected with Myc‐ZNF8, Flag‐SMYD3 and Flag‐tagge SMYD3 domains MYND, SET and CTD (left panel). Co‐immunoprecipitation with anti‐Myc antibody in HEK293T cells co‐transfected with Flag‐SMYD3, Myc‐ZNF8, Myc‐tagged ZNF8 domains KARB, Linker, ZF repeats and C. Lower panel represents a schematic representation of ZNF8 mutants (right panel). F) RT–qPCR analysis of signature genes in control and ZNF8 knockout MDA‐MB‐231 cells with SMYD3 overexpression (n ≥ 3). G) Representative images and quantification of Transwell assay for migration and invasion in control and ZNF8 knockout MDA‐MB‐231 with SMYD3 overexpression (n ≥ 3). For A, F, and G, data represent mean ± SD, and significance was determined with the Student's t test, ns, *p* > 0.05; *, *p* < 0.05; **, *p* < 0.01; ***, *p* < 0.001; ****, *p* < 0.0001.

ZNF8 is a member of the Krüppel‐associated box domain zinc finger proteins (KRAB‐ZFPs), which have been demonstrated to function through the recruitment of transcriptional regulators and mediators of histone epigenetic modification.^[^ ^18^ ^]^ To dissect the mechanism underlying ZNF8‐mediated transcriptional activation, the impact of ZNF8 on histone modification in the promoter regions of lung metastasis signature genes was investigated. ZNF8 overexpression led to an increase in the H3K4me3 level of target genes (**Figure** **7**A) but did not have a notable impact on the levels of other epigenetic modifications of gene promoters, such as H3K27ac, H3K16ac, H3K36me2, and H3K27me3 (Figure S8A, Supporting Information). These results suggested that ZNF8 specifically regulates the H3K4me3 epigenetic modifications at lung metastasis signature genes, but the underlying mechanism remains unclear. Different histone posttranslational modification enzymes modify specific histone marks, and SMYD3 is a histone 3 lysine‐4 (H3K4) methyltransferase that can promote H3K4me3 modification in gene promoters.^[^ ^19^ ^]^ Recently, Fenizia et al reported that SMYD3 could activate gene transcription through interactions with Smad3 during TGF‐β‐induced EMT,^[^ ^20^ ^]^ but it is still unknown whether SMYD3 could regulate the lung metastasis through TGF‐β pathway. Therefore, we firstly studied the regulation of SMYD3 on the promoter region of signature genes, and the results revealed that overexpressing SMYD3 markedly increased and inhibiting SMYD3 with BCI121 decreased the H3K4me3 at the MDA‐MB‐231 gene promoter (Figure S8B, C, Supporting Information). Moreover, our results revealed that SMYD3 could upregulate the expression of target genes and the invasion and metastasis ability of breast cancer cells, while TGF‐β inhibitor SB431542 could significantly inhibit these promoting effects of SMYD3 (Figure S8D, E, Supporting Information). Meanwhile, we also knocked down other H3K4 methyltransferases such as SETD7, MLL1, MLL3^[^ ^21^ ^]^ to examine their regulatory effects on the promoter and expression of signature genes, while the results revealed that the promoting effect of ZNF8 on the H3K4me3 level in the promoter and the expression of signature genes was not depending on these H3K4 methyltransferases (Figure S9A,B, Supporting Information).

Considering that SMYD3 was reported to interact with Smad3, we wonder if there is ZNF8/SMYD3/Smad3 binding. Therefore, the Co‐IP experiments were performed with the ZNF8 antibodies. Indeed, the results revealed that ZNF8 could bind both SMYD3 and Smad3 (Figure 7B; Figure S10A, Supporting Information), and confocal fluorescent images showing the colocalization of ZNF8, Smad3 and SMYD3 in the nuclear of MDA‐MB‐231 cells (Figure S10B, Supporting Information). Our results also showed the activation of the TGF‐β pathway enhanced the interactions between SMYD3 and Smad (Figure S10C, Supporting Information). Considering the enhanced interaction between ZNF8 and Smad3 after treated with TGF‐β1 (Figure 1H), these results collectively indicated TGF‐β stimulation enhanced ZNF8/SMYD3/Smad3 binding. Additionally, a GST pull‐down assay confirmed the direct physical interactions among ZNF8, Smad3, and SMYD3 (Figure 7C). Furthermore, we investigated whether ZNF8 is essential for the interaction between Smad3 and SMYD3. Interestingly, our results revealed that the interaction between Smad3 and SMYD3 was significantly suppressed when ZNF8 was knocked out in MDA‐MB‐231 cells (Figure 7D; Figure S10D, Supporting Information). Next, we mapped the mutual interaction regions in ZNF8 and SMYD3 and found that the C terminal domain (CTD) of SMYD3 interacted with the zinc finger (ZF) domain of ZNF8 (Figure 7E).

Based on these results, we propose that ZNF8 regulates the TGF‐β/Smad3 pathway by recruiting SMYD3.Consistent with these findings, ZNF8 significantly enhanced the binding of SMYD3 to the promoter regions of the lung metastasis signature genes (Figure S11A, Supporting Information), while the promoting effect of SMYD3 to the H3K4me3 level of signature genes was notably reduced by ZNF8 knockout (Figure S11B, Supporting Information). Moreover, the effect of SMYD3 overexpression on the transcription of lung metastasis signature genes was significantly suppressed by ZNF8 knockout (Figure 7F). Furthermore, ZNF8 knockout significantly inhibited the ability of SMYD3 to promote cell migration and invasion (Figure 7G). Therefore, our results confirmed that ZNF8 promoted the transcriptional activation of lung metastasis signature genes by recruiting SMYD3 subsequently promoting breast cancer metastasis.

### 2.8 Targeting SMYD3 Inhibits ZNF8‐Mediated Breast Cancer Metastasis

**Figure 8:**
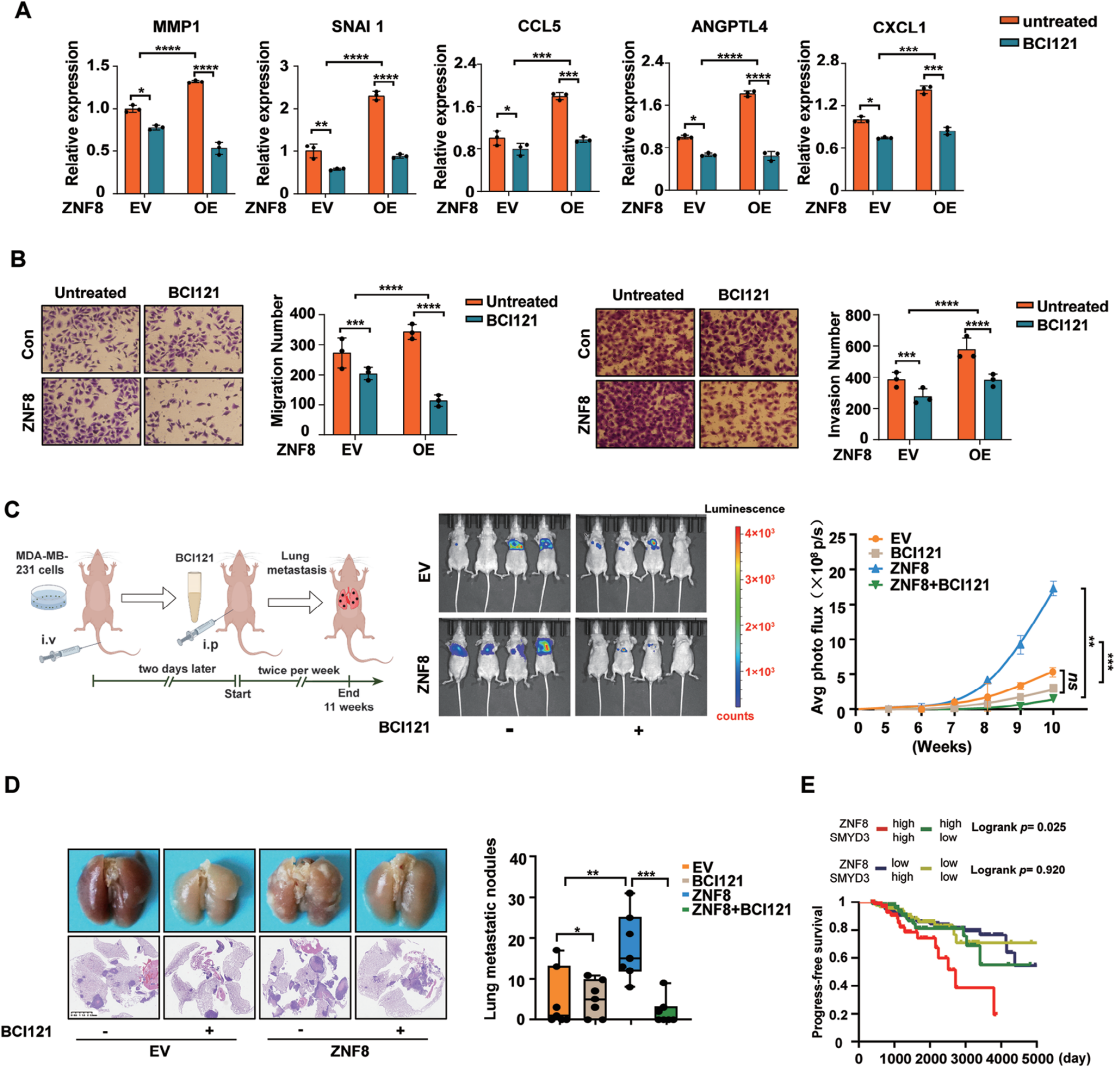
Targeting SMYD3 inhibits ZNF8‐mediated breast cancer metastasis. A) RT–qPCR analysis of signature genes in ZNF8‐overexpressing MDA‐MB‐231 cells with BCI121 treatment (n ≥ 3). B) Representative images and quantification of Transwell assay for migration and invasion in ZNF8‐overexpressing MDA‐MB‐231 cells with BCI121 treatment (n ≥ 3). C) Schematic diagram of the study of nude mice lung metastasis via tail‐vein injection of MDA‐MB‐231 breast cancer cells and treated with BCI121, (n = 6 mice per group) (left panel). Lung metastasis was analyzed by in vivo bioluminescence imaging; plot represents normalized photon flux from mouse lungs (right panel). D) Representative images and quantification of lung metastatic nodules in lung tissues harvested at Week12, (n = 6 mice per group). E) Kaplan‐Meier progress‐free survival stratified by high ZNF8 and SMDY3 (red), high ZNF8 and low SMYD3 (green), low ZNF8 and high SMDY3(blue), low ZNF8 and SMYD3 (yellow) mRNA expression in TCGA Breast cancer cases, (n = 885). Log–rank (Mantel–Cox) test. For A‐D, data represent mean ± SD, and significance was determined with the Student's t test, ns, *p* > 0.05; *, *p* < 0.05; **, *p* < 0.01; ***, *p* < 0.001; ****, *p* < 0.0001.

Our studies confirmed that ZNF8, which interacts with Smad3, plays a critical role in the regulation of breast cancer metastasis via TGF‐β through SMYD3. Therefore, targeting this mechanism might have significant implications for the inhibition of breast cancer metastasis. BCI121 is a small molecule inhibitor of the histone methyltransferase SMYD3 that effectively inhibits SMYD3 function.^[^ ^22^ ^]^ BCI121 significantly suppressed the ability of ZNF8 to promote the expression of lung metastasis signature genes in MDA‐MB‐231 cells, and this inhibitory effect was more pronounced in ZNF8‐overexpressing cells (**Figure** **8**A), regardless of the TGF‐β pathway activation or not (Figure S12A, Supporting Information). BCI121 also significantly inhibited the effects of ZNF8 on both migration and invasion in MDA‐MB‐231 cells, and cells overexpressing ZNF8 were more sensitive to BCI121 (Figure 8B). Furthermore, treatment with BCI121 effectively suppressed the ZNF8‐induced increase in the metastatic potential of breast cancer cells (Figure 8C,D). Additionally, the inhibitory effects of BCI121 on the transcription of lung metastasis signature genes, cell migration, and invasion were nearly eliminated in ZNF8 knockout breast cancer cells (Figure S12B, C, Supporting Information). Moreover, BCI121 significantly suppressed the spontaneous lung metastasis of breast tumors in the transgenic animal study, but this effect was also absent in ZNF8 conditional knockout mice (Figure S12D, Supporting Information). These results further suggested the critical role of the ZNF8‐SMYD3 axis in breast cancer metastasis.

With respect to clinical data, we analyzed the relationship between SMYD3 expression levels and breast cancer prognosis using the TCGA database and observed that there was no significant association between SMYD3 expression and progression‐free survival (PFS) or OS (Figure S12E, Supporting Information). However, in the subgroup analysis, we found that the prognosis of patients with high ZNF8 and SMYD3 expression was significantly worse than that of patients in the other three groups (ZNF8 high‐expression and SMYD3 low‐expression; ZNF8 low‐expression and SMYD3 high‐expression; ZNF8 and SMYD3 both low‐expression). Moreover, SMYD3 expression was significantly negatively correlated with poor breast cancer prognosis in patients with high ZNF8 expression but not in patients with low ZNF8 expression (Figure 8E; Figure S12F, Supporting Information). Our results confirmed that targeting SMYD3 can inhibit ZNF8‐mediated invasion and metastasis of breast cancer cells, indicating that targeting the mechanism by which ZNF8 recruits SMYD3 may be an effective strategy for the TGF‐β‐mediated lung metastasis of breast cancer.

## 3 Discussion

In this study, we screened in human breast cancer cells and identified the transcription factor ZNF8 as a novel protein that interacts with Smad3, which is a well‐known mediator of the TGF‐β pathway. ZNF8 binds to Smad3, and promotes multiple processes such as cell migration and invasion, EMT, endothelial cell adhesion, and vascular invasion by enhancing the transcription of lung metastasis signature genes. More importantly, ZNF8 knockout markedly inhibits neutrophil infiltration in the lung, which might establish a premetastatic niche for tumor cells.^[^ ^23^ ^]^ Additionally, we demonstrated that ZNF8 is indispensable for TGF‐β pathway‐promoted lung metastasis. Mechanistically, ZNF8 recruits the histone methyltransferase SMYD3 to interact with Smad3, which jointly increases the H3K4me3 epigenetic modifications of the target genes promoter. This, in turn, facilitates the transcriptional activation of lung metastasis signature genes, ultimately leading to lung metastasis in breast cancer.

The TGF‐β pathway has been reported to participate in the metastasis cascades by promoting EMT, regulating the immune response, remodeling the extracellular matrix, and promoting angiogenesis.^[^ ^24^ ^]^ Currently, TGF‐β‐targeted therapies have shown promising inhibitory effects on metastasis.^[^ ^25^ ^]^ However, due to the pleiotropic effects of TGF‐β, systemic blockade of TGFβ may lead to adverse effects such as liver toxicity and cardiotoxicity, limiting the clinical potential of such drugs.^[^ ^26^ ^]^ Therefore, it is necessary to explore the specific regulator of TGF‐β pathway involved in the process of metastasis to achieve more precise medical treatment. Smad3 mediates the functions of the TGF‐β pathway by interacting with various cofactors, and these R‐Smad binding partners include transcription factors from the forkhead, homeobox, bHL and AP1 families.^[^ ^27^ ^]^ However, the Smad3 cofactors involved in lung metastasis in breast cancer are still poorly understood. In this study, we initially conducted proteomic analysis of Smad3‐interacting proteins in breast cancer cells and elucidated the functional clustering of these proteins. The analysis demonstrated that proteins involved in transcriptional regulation were significantly enriched, which was in accordance with a previously reported study in human pluripotent stem cells,^[^ ^28^ ^]^ suggesting that these functions of Smad3 are evolutionarily conserved. After further analysis of transcription factors, ZNF8 was identified because of its significant correlation with the prognosis of patients with breast cancer. Furthermore, the results revealed that ZNF8 mediated increases in the expression of lung metastasis signature genes depend on interactions with Smad3. Thus, our findings suggest that ZNF8 acts as a selective coactivator of the TGF‐β/Smad3 pathway, specifically facilitating lung metastasis in breast cancer while not impacting tumor cell proliferation. This highlights the potential of targeting the TGF‐β pathway through ZNF8 as a promising strategy for addressing breast cancer lung metastasis.

Recent studies have also demonstrated that the infiltration of neutrophils in distant organs also plays a role in this process of metastasis, as it facilitates the creation of a microenvironment that is favorable for tumor colonization, commonly referred to as the premetastatic niche.^[^ ^29^ ^]^ Notably, our results showed that ZNF8 could specifically induce neutrophil infiltration, particularly in early metastatic organs, by promoting the transcriptional activation of neutrophil chemokines. The formation of neutrophil extracellular traps (NETs) has been reported playing important role in promoting metastasis of tumor cells. Besides NETs, degranulation of neutrophil also could enhance metastatic colonization of cancer cells.^[^ ^30^ ^]^ Of note, our results showed that ZNF8 expression was positively correlated with neutrophil degranulation, which indicated effect of ZNF8 on neutrophil degranulation might participate in the promoting metastasis function of ZNF8. However, the specific mechanism should be explored in the future. Additionally, ZNF8 is a member of the KRAB‐ZFPs (KRAB‐type zinc finger proteins) family, which is the largest family of transcription factors/transcriptional regulators in mammals and is reportedly correlated with several human tumors.^[^ ^31^ ^]^ The enhancement of neutrophil infiltration in lung metastasis by ZNF8 expands the understanding of the roles of KZNF family transcription factors in the tumor immune microenvironment. However, further studies are needed to ascertain the importance of neutrophil infiltration induced by ZNF8 in metastatic organs, such as the bones, liver, and brain.

The KRAB‐ZFPs family is characterized by the conserved KRAB domain at the N‐terminus and multiple C2H2‐type zinc finger structures in the middle and C‐terminus that recognize specific DNA sequences.^[^ ^32^ ^]^ The function of most KRAB‐ZFPs is unknown, but a few have been demonstrated to form transcriptional complexes by recruiting the transcriptional regulators KAP1 or SETDB and function mainly as transcriptional repressors.^[^ ^33^ ^]^ Our previous research also showed that ZNF498 can act as a p53 corepressor in liver cancer cells.^[^ ^34^ ^]^ This study proposed another mode of action for KRAB‐ZFPs by which ZNF8 acts as a coactivator of Smad3 and recruits the epigenetic regulator SMYD3 to promote H3K4me3 modification, thereby activating the transcription of lung metastasis signature genes in breast cancer, which has not been previously reported.

Although previous studies have reported the regulation of epigenetic modifications by KRAB‐ZFPs family members in the TGF‐β pathway,^[^ ^35^ ^]^ there are few targeted therapeutic strategies for metastasis. In this study, we attempted to target the ZNF8–SMYD3 axis by using BCI121, an inhibitor of SMYD3. BCI121 indeed significantly suppressed ZNF8‐mediated invasion and metastasis of breast cancer cells, which confirmed that the ZNF8–SMYD3 axis is a good candidate target for the prevention and treatment of breast cancer lung metastasis. Considering that SMDY3 inactivation under normal conditions does not lead to detectable phenotypes, so the SMYD3 inhibitors have less side effects compared with TGF‐β pathway inhibitors. Moreover, our clinical studies suggested that the prognostic significance of SMYD3 in breast cancer is influenced by ZNF8 expression, further highlighting the key role of ZNF8 in this axis. A follow‐up study to screen for inhibitors targeting ZNF8 is underway in our laboratory.

## 4 Conclusion

In conclusion, we identified a novel prometastasis TGF‐β/Smad3 cofactor in breast cancer. We also demonstrated that ZNF8, a Smad3‐interacting protein, is indispensable for multiple processes related to lung metastasis in breast cancer by epigenetic modulation. These findings provide valuable insights into the molecular mechanisms by which TGF‐β pathway signaling induces breast cancer lung metastasis and suggest a potential therapeutic target for the treatment.

## 5 Experimental Section

### Cell Culture

MCF‐7, ZR‐75‐1, MDA‐MB‐231, and Hs578T human breast cancer cell lines and human umbilical vein endothelial cells (HUVEC), human embryonic kidney 293T (HEK‐293T) cells from the American Type Culture Collection (ATCC) were obtained. MCF‐7, MDA‐MB‐231, Hs578T, and HEK‐293T cells were cultured in high glucose DMEM (Gibco; 11965‐092) supplemented according to ATCC guidelines. RPMI‐1640 (Gibco; 61870‐036) and F‐12K Medium (ATCC; 30–2004) supplemented with ATCC guidelines were used to culture ZR‐75‐1 cells and HUVEC, respectively. All cell lines were cultured in the indicated medium with 10% fetal bovine serum (Gibco; 10 099 141) and a 1% Antibiotic–Antimycotic (Gibco; 15‐240‐062) following ATCC's instructions. The cells were maintained at 37 °C and 5% CO2 and were confirmed to be mycoplasma‐free with MycoAlert (Lonza; LT07‐418)_._ After culturing for 24 h in complete medium, conditioned media (CM) from ZNF8 overexpressing MCF‐7 cells or ZNF8 knockout MDA‐MB‐231 cells were collected as specified for each experiment,

### Anti‐Smad3‐Immunoprecipitation and LC‐MS/MS

MDA‐MB‐231 nuclear lysates were extracted using a nuclear and cytoplasmic protein extraction kit from 2 × 10^8^ cells and centrifuged at 100 000 g for 30 min at 4 °C. For immunoprecipitation, nuclear lysates were incubated with 1 µg anti‐Smad3 antibodies or normal IgG for 3 h, and 30 µL protein A/G PLUS agarose was later added and incubated for 8 h. Agarose was collected by centrifugation at 3000 g for 2 min at 4 °C and washed with NETN lysis buffer 3–4 times for 5 min. Precipitated protein was resolved in 2 × protein loading buffer and boiled for 10 min. The protein was loaded onto SDS‐PAGE, immunoblotted with the indicated antibody, and stained with Coomassie blue. The full lanes were cut into small bands regardless of whether the protein band was visible, and were subjected to LC‐MS/MS sequencing and data analysis as previously described. The mass spectrometry results were searched by MaxQuant, and the data quality control was performed based on the protein group file of the results. The quantitative results were scored using SAINT express for interaction confidence (SAINT score ≥ 0.9), and a total of 626 highly confident interacting proteins were identified. The highly confident interactions included 66 transcription factors reported in the transcription factor database Animal TFDB and the interaction network was drawn using Cytoscape.

### Western Blots and Immunoprecipitation

For preparing the protein extracts for western blots, samples were mixed with Laemmli loading buffer containing Tris‐HCl (0.1 M, pH 7.0), SDS (4%), glycerol (20%), DTT (1 mM), and protease inhibitors (Roche, 4 693 116 001). The mixtures were then loaded onto SDS polyacrylamide gels and separated by electrophoresis. The separated proteins were transferred to NC membranes (Pall, C05‐05002), which were then probed with specific antibodies. The immunoblots were visualized using the Bio‐Rad system. Antibody information is provided in Table S3 (Supporting Information). For immunoprecipitation experiments, cells were lysed in a buffer containing KCl (200 mM), Tris‐HCl (20 mM, pH 7.9), MgCl_2_(5 mM), glycerol(10%), EDTA(0.2 mM), and NP‐40(0.1%), supplemented with protease inhibitors (Roche, 4 693 116 001). The lysates were then incubated with specific antibodies or control IgGs overnight at 4 °C. The beads‐bound immunoprecipitates were washed and eluted in Laemmli loading buffer for western blot analysis.

### GST‐Pull Down Assay

In the GST‐pull down experiment, 1 µg of GST, GST‐ZNF8, or GST‐Smad3, protein was immobilized on Glutathione‐Sepharose 4B (GE, 17 075 601). This was then incubated with 6× His‐tagged Smad3 or SMYD3 protein purified from bacterial culture for 2 hours at 4 °C in a buffer containing KCl(120 mM), Tris‐HCl (20 mM, pH 7.9), MgCl2 (5 mM), EDTA(0.2 mM), glycerol (10%), NP‐40 (0.2%), and protease inhibitors (Roche, 4 693 116 001). After incubation, the beads were washed and then analyzed by western blotting.

### Patient Specimens and Immunohistochemistry Staining

The Qilu cohort 1 involved 152 patients with primary breast cancer, and the metastasis was analyzed using Kaplan‐Meier on samples with follow‐up information (Table S1, Supporting Information). The Qilu cohort 2 involved 108 patients with different status of metastasis (Table S2, Supporting Information), and the Qilu cohort 3 involved 49 patients with metastatic lymph nodes. All samples were obtained including another four primary tumors and paired lung metastatic tumors from Qilu Hospital of Shandong University with consent from all subjects and approval from the hospital's institutional research ethics committee. The expression levels of ZNF8 were semi‑quantified using a semi‑quantitative IHC scoring system, as previously described.^[^ ^36^ ^]^ The percentage of positive tumor cells was graded on a scale between 0 and 4, as follows: 0, none; 1, 1–10%; 2, 11–50%; 3, 51‑80%; 4, >80%. The intensity of staining was graded on a scale between 0 and 3, as follows: 0, none; 1, weak staining; 2, moderate staining; 3, strong staining. The combination of the extent (E) and intensity (I) of staining was obtained as the product of E and I (EI), which varied between 0 and 12 for each sample. Using the X‑tile software program (version 3.6.1; The Rimm Lab, Yale University; https: //medicine.yale.edu/lab/rimm/research/software.aspx), a significant cutoff point for ZNF8 was identified in terms of distant metastasis‐free survival (DMFS) in patients with breast cancer. A cutoff score of 6 was selected, and the score≥6 was considered high expression. The Declaration of Helsinki of 1975 was followed in all research.

### CRISPR/Cas9 Experiments

MDA‐MB‐231 and Hs578T cells were infected by pLentiCRISPR v2 (Addgene) with either human ZNF8 sgRNA or negative control sgRNA vector for deletion of ZNF8. After 48 h, separated cells were plated on 96‐well plates and treated with Puromycin for 2 weeks at 2.0 g mL^−1^. Then the viable colonies were expanded and immunoblots were performed to verify the ZNF8 levels. The oligo sgRNA sequences used are listed in Table S4 (Supporting Information).

### Lentiviral Overexpression

The ZNF8 (Flag‐tagged) cDNA was cloned into the pLV‐Neo‐ZNF8 lentivirus vector, and then the ZR‐75‐1, MCF‐7, and MDA‐MB‐231 cells were either infected with 1 µg ZNF8 overexpression plasmid or with an empty control vector. Once infected, the cells were treated with G418 (Millipore, 345 810) for two weeks to obtain stable ZNF8 overexpression. The ZNF8 expression was verified by immunoblotting.

### siRNA Knockdown

The siRNAs were transfected into cells using PE according to the manufacturer's instructions. GenePharma Company (Shanghai) provided oligo siRNAs for transfection, and the siRNA sequences are listed in Table S5 (Supporting Information).

### Cell Proliferation and Colony‐Forming Assay

The viability of the cells was determined using the Cell Counting Kit‐8 (Dojindoe, CK04) according to the manufacturer's instructions. Cells were seeded and cultured (2 × 10^3^ per well in 100 µL of medium) in 96‐well microplates. After 24 h, 10 µL of CCK‐8 reagent was added to each well and then cultured for 2 h. With the SpectraMax microplate reader (Molecular Devices, 340PC384), the proliferation of cells was estimated based on the absorbance at 450 nm using wells without cells as blanks. The relative cell viability was expressed as percentage of that of the control cells.

For the colony‐forming assay, trypsinzed cells were dispensed into six‐well tissue culture dishes with a cell density of 300 per well. Cells were fixed and stained with Giemsa to visualize colonies at the end of a 14‐day drug‐free culture. Experiments were performed in triplicate.

### Immunofluorescence

Cells seeded in confocal dishes were fixed with 4% paraformaldehyde and then kept stable for 10 min to rupture the cell membranes with 0.2% Triton. To block non‐specific antigen‐binding sites, 30 min of BSA treatment was performed following three PBS washings. After incubating the primary antibodies diluted in the same blocking buffer overnight at 4 °C, the secondary antibodies were incubated for 1 hour at room temperature. The images were taken either with fluorescence microscope (Nikon, Ts2‐FL) or confocal fluorescence microscope (ZEISS, LSM800).

For the multiplex IF staining assays to determine the subcellular localization and co‐localization of ZNF8, Smad3, and SMYD3 in MDA‐MB‐231 cells. Cells seeded in confocal dishes were sequentially stained with primary antibodies and HRP‐conjugated secondary antibodies. One of the three dye reagents was used for staining, followed by microwave treatment and another round of staining. Anti‐ZNF8, anti‐SMYD3, and anti‐Smad3 were used as primary antibodies. Dyes PDD520, PDD570, and PDD650 (Panovue, Beijing,China) were used for staining.

For neutrophil infiltration assay of murine tissues, the paraffin‐embedded tissue sections underwent a process of dewaxing and rehydration, followed by a critical step to block endogenous peroxidase activity. To enhance antigen visibility, a high‐temperature antigen retrieval protocol was employed. Subsequently, the sections were processed further for immunofluorescence (IF). The sections were incubated for overnight at 4 °C with the anti‐Ly6G primary antibodies. Subsequently, the sections were incubated with Alexa Fluo488 Goat anti‐Rabbit IgG Cross‐Adsorbed secondary antibody. Nuclei were counterstained with 4′‐6′‐diamidino‐2‐ phenylindole (DAPI). Images were captured using a confocal laser‐scanning microscope (ZEISS, LSM800).

### Transwell Migration and Invasion Assay

Transwell migration and invasion assays were performed using a Transwell chamber (Corning, 354 480) with or without a Matrigel‐coated filter. The 3 × 10^4^ MDA‐MB‐231 cells/1.5 × 10^4^ Hs578T cells /6 × 10^4^ ZR‐75‐1 cells/8 × 10^4^ MCF7 cells/8 × 10^4^ tumor cells isolated from lung metastasis lesion of WT and ZNF8‐KO mice suspended in 200ul serum‐free medium were plated on the upper chamber membranes. The insert was incubated in 600 µL medium with 20% FBS for 24 h at 37 °C, and the migrating or invading cells were fixated with 70% ethanol for 15 min and stained with 2% Crystal Violet for 10 min at room temperature. The cell count was performed with Olympus digital camera (Olympus, DP71).

### Endothelial Permeability

The passage of rhodamine‐conjugated dextran (70 kDa) (Sigma, R9379) was used to determine the permeability of treated HUVEC monolayers on Transwell filters (0.4‐µm pore size) (Corning, 3396). Briefly, the top well was added with 20 mg/ml rhodamine‐dextran, and then the fluorescence in the bottom well was monitored by measuring 40 µL medium aliquots with the SpectraMax microplate reader (Molecular Devices, 340PC384) at 544 nm excitation and 590 nm emission in a time course.

For in vivo assay, the murine lung tissues were cleared of blood by perfusing with 50 ml of PBS through the right ventricle. The tissues were then rinsed with pre‐chilled PBS, fixed them in 4% PFA for 2 hours at 4 °C on a shaker. After that, the fixed tissues were dehydrated overnight with 30% sucrose in PBS, and embedded in OCT (Sakura 4583) for 1 h at 4 °C, and frozen at −80 °C. The tissues were sectioned to 10 mm thickness, and observed with confocal fluorescence microscope (ZEISS, LSM800)

### Tumor Cell–Endothelial Cell Binding Assay

HUVECs were seeded in 24‐well plates and incubated for 48 hours to achieve a uniform monolayer. Before adhering to the endothelial cell monolayer, tumor cells were labeled with CFDA‐SE (Beyotime, C1031) at a concentration of 5 mg mL^−1^ for 10 min at 37 °C. The wells were then washed twice with PBS, and images were captured using a fluorescence microscope.

### Animal Studies

Female mice were used because mammary cancers occur primarily in females. Pathogen‐free female Balb/c nude mice and C57BL/6 4–6 weeks old were used for lateral tail vein injection. To investigate lung metastasis formation, 5 × 10^5^ viable MDA‐MB‐231 cells were collected in PBS and then injected into the lateral tail vein in a volume of 0.1 ml. The endpoint assays were conducted at 15 weeks post‐injection, unless early termination was necessary due to significant morbidity. For the TGF‐β inhibition experiment, mice were treated with the SB431542 (1 µM, 100uL per mouse) by intraperitoneal injection twice per week. For neutrophil depletion experiments, anti‐Ly6G (200 µg per mouse, clone 1A8), and isotype controls mouse IgG2a (clone 2A3) were injected intraperitoneally. Treatment was administered on day −1 when tumor cell injection with 4 × 10^6^ viable ZNF8 overexpression and empty vector 4T1cells, and thereafter every 3 days until experimental endpoint. For orthotopic metastasis studies, 4–6 weeks old female NOG mice were purchased from Charles River (Beijing) and the tumor cells were then resuspended in a 50:50 solution of PBS and Matrigel at a concentration of 4 × 10^7^ cells mL^−1^. a mini incision was made to expose the mammary gland after the mice were anesthetized, and 1 × 10^6^ cells (50 uL) were injected directly into the mammary fat pad. The primary tumor outgrowth was monitored weekly by measuring the tumor length (L) and width (W), and the tumor volume was calculated as πLW^2^/6. For metastasis assays, tumors reaching a volume greater than 300 mm^3^ were surgically resected at 7 weeks and the bioluminescent imaging was used to monitor the development of metastases after resection. In order to visualize and analyze bioluminescent signals, mice were anesthetized and injected intraperitoneally with 1.5 mg of D‐luciferin (15 mg/ml^2^ in PBS) (Perkinelmer, K9937PE). 10 and 12 min later after injection, the imaging was completed using a Perkinelmer IVIS Spectrum system (Perkinelmer, 124 262) coupled to Living Image analysis software. After H&E staining of the lung, metastatic nodules on its surface were counted under dissecting microscope. During the study, all mice were housed and handled according to protocols approved by the institutional Animal Care and Use Committee of National Center for Protein Sciences (Beijing).

### RNA Isolation and Quantitative RT‐PCR

Total RNA was isolated using Trizol reagent (Sigma, T9424), and cDNA was generated from 500 ng of total RNA using a ReverTra Ace qPCR RT Kit (TOYOBO, FSQ‐101) following the manufacturer's protocol. Subsequently, fluorescence quantitative PCR was carried out using the SYBR Green Realtime PCR Master Mix (TOYOBO, QPK‐201) in a Bio‐Rad detection system. Three technical replicates were carried out for all quantitative PCR. The sequences of the primers were listed in Table S4 (Supporting Information).

### RNA Sequencing

For RNA‐seq of ZNF8 knockout and control MAD‐MB‐231 cells, cells were cultured as described above. For RNA‐seq of TGF‐β pathway, the MDA‐MB‐231 cells were treated with 5uM SB431542 for overnight. Cells were first lysed with h TRIzol reagent (Sigma, T9424) and then purified with the Mini‐RNeasy kit (Qiagen, 74 106). RNA sequencing libraries were made, and samples were sequenced on an Illumina HiSeq 2000 by Novogene (Beijing). Hisat2 v2.0.5 was used to build the reference genome index, and align clean paired‐end reads to the reference genome. Read counts mapped to each gene were calculated using FeatureCounts v1.5.0‐p3. And then the FPKMs were calculated using the length and read count mapped to each gene. According to the negative binomial distribution, an analysis of differential expression was performed with DESeq2. GO and KEGG enrichment analyses were conducted based on the results of comparisons.

### In Vivo Lung Permeability Assays

For the assays of lung blood vessel permeability in vivo, tumor cells with indicated treatment were injected into the lateral tail vein. After inoculation, mice were injected intravenously with rhodamine‐conjugated dextran (70 kDa) (Sigma, R9379) at 2 mg per 20 g body weight one day later. The mice were sacrificed after 3 h, and 5 mL of PFA 4% was injected intratracheally to fix the lung before the extracting. Following that, the lungs were frozen and 10 mm sections were taken to be examined under a confocal fluorescence microscope for the presence of vascular leaks.

### Transgenic Mouse Models

Female mice were used because mammary cancers occur primarily in females. Murine mammary cancer model C57BL/6JBL/6J‐Tg (MMTV‐PyMT) was gifted by Professor Lingqiang Zhang (State Key Laboratory of Proteomics, Beijing Proteome Research Center, National Center for Protein Sciences Beijing, Beijing Institute of Lifeomics). The Znf8^fl/fl^ mice were obtained from Cyagen (Guangzhou, China) through its transgenic animal services. MMTV‐Cre/Znf8^fl/fl^ animals were then bred with mice carrying the PyMT transgene to get female cohorts of Znf8^fl/fl^ ‐PyMT (WT), Znf8^fl/fl Cre^‐PyMT (KO) and heterozygous knockout Znf8 ^fl/+ Cre^‐PyMT (Het) mice. The mice were aged, and mammary tumor development was monitored. At the age of 5 weeks, mice were randomly assigned to two experimental groups to study tumor formation and lung metastasis. Mice were treated with the BCI121 (1 mM kg^−1^) by intraperitoneal injection twice per week. All mice were killed after 23 weeks and lung metastasis was examined. After staining with H&E, the lung metastatic nodules were counted using a dissecting microscope. During the study, all mice were housed and handled according to protocols approved by the institutional Animal Care and Use Committee of National Center for Protein Sciences (Beijing).

### Flow Cytometry

For flow cytometry, lung and primary tumor samples from ZNF8 KO mice and control WT group were dissociated into single‐cells suspensions with Hank's Enzyme Free Cell Dissociation Solution (EMD Millipore, S‐004‐C) and filtered through a 70‐µm strainer. The single‐cell suspensions were stained with anti‐CD16/32 in a staining solution at 4 °C for 30 min, then stained with appropriate antibody (Table S3, Supporting Information), and the percentage of CD3^+^ T cells, CD19^+^ B cells, CD11b^+^F4/80^+^ macrophages, CD11c^+^MHCII^+^ DCs, CD11b^+^Ly6G^+^ neutrophils were evaluated using the BD LSRFortessa SORP (BD Biosciences).

### LC‐MS/MS for Proteomics Analysis

For proteomic experiments, lung samples from ZNF8 KO mice and control WT group were sliced into small pieces (5 µm per piece). To remove the bulk of the paraffin, FFPE rolls underwent preheating in an oven at 65 °C for 10 min and fully deparaffinized using xylene, concentration gradient ethanol (100%, 90%, 75%), and water followed by air dry. Sliced FFPE specimens were re‐suspended in tenfold volume (200 µL per 5 pieces) of lysis buffer containing 300 mM Tris‐HCl (pH 8.6), 1% (w: v) sodium deoxycholate, and EDTA‐free protease cocktail inhibitors. Mass spectrometry (MS) samples were prepared and analyzed as previously described.^[^ ^37^ ^]^

### ChIP‐qPCR

3 × 10^7^ MDA‐MB‐231 cells were collected and washed once in PBS. The cells were then cross‐linked with 1% formaldehyde for 10 minutes at room temperature, which was quenched by addition of 0.125 M final glycine for 5 minutes at room temperature. Chromatin immunoprecipitation, including sample preparation, sonication, immunoprecipitation by specific antibodies (Table S3, Supporting Information) and purification was conducted as previously described.^[^ ^38^ ^]^ Quantification of ChIP products was performed with SYBR Green Realtime PCR Master Mix (TOYOBO, QPK‐201) in a Bio‐Rad detection system and normalized to those obtained with a nonimmune serum (IgG). % of DNA inputs are used to express the data. The primers for ChIP–qPCR are detailed in Table S4 (Supporting Information).

### Statistical Analysis

Student's t test, two sided, was used for all two‐sample statistical analyses and the results were presented as the mean ± SD. Pearson χ2‐test was applied to analyze the relationship between ZNF8 expression and clinicopathological characteristics. The online database, Kaplan‐Meier plotter (http://kmplot.com/analysis/), which was constructed based on microarray and RNA‐seq data from databases such as GEO, EGA, and TCGA, and encompassing the most comprehensive information from 6234 breast cancer patients, was adapted to evaluate the prognostic value of top ten Smad3 binding transcription factors on OS and DMSF for breast cancer patients. The mRNA expression data of ZNF8 and SMYD3 for breast cancer was downloaded from The Cancer Genome Atlas (TCGA). The Log‐rank (Mantel–Cox) test was used to analyze the survival data for breast cancer patients (follow‐up time for at least a year) and animal study with GraphPad Prism. GraphPad Prism was also used to perform significance analyses for RT‐qPCR, ChIP‐qPCR, IHC quantification, HUVEC adhesion, and Transwell assays. GSEA was performed with GSEA software (http://software.broadinstitute.org/gsea/index.jsp). Statistics are shown on the graphs with *p* values and significance was determined by *p* < 0.05.

### Ethics Approval Statement

We obtained all samples from Qilu Hospital of Shandong University with consent from all subjects and approval from the hospital's Institutional Research Ethics Committee. The animal operations in this study were evaluated and approved by the Institutional Animal Care and Use Committee of National Center for Protein Sciences (Beijing).

## Conflict of Interest

The authors declare no conflict of interest.

## Author Contributions

T.C. and G.W. designed the research studies. G.W., A.J., D.K., Z.H., and Z.X. conducted the experiments. G.W. prepared the figures and drafted the manuscript. G.W., A.J., and D.K. participated in the analysis and interpretation of data. L.Y. performed PPI analysis. Z.X., X.R., L.Y., H.S., S.H., and Y.W. provided technical and/or material support. T.C., G.H., and W.J. supervised the project and revised the manuscript. All authors have read and approved the article.

## Supporting information

Supporting Information

Supporting Information

## Data Availability

The data that support the findings of this study are available from the corresponding author upon reasonable request.

## References

[1] a) S. Loibl , P. Poortmans , M. Morrow , C. Denkert , G. Curigliano , Lancet 2021, 397, 1750; 33812473 10.1016/S0140-6736(20)32381-3

[2] B. Medeiros , A. L. Allan , Int. J. Mol. Sci. 2019, 20, 2272.31071959

[3] M. Yousefi , R. Nosrati , A. Salmaninejad , S. Dehghani , A. Shahryari , A. Saberi , Cell Oncol (Dordr) 2018, 41, 123. 29568985 10.1007/s13402-018-0376-6 PMC12995240

[4] K. Ganesh , J. Massague , Nat. Med. 2021, 27, 34. 33442008 10.1038/s41591-020-01195-4 PMC7895475

[5] M. K. Ibragimova , M. M. Tsyganov , E. A. Kravtsova , I. A. Tsydenova , N. V. Litviakov , Int. J. Mol. Sci. 2023, 24, 15625. 37958607 10.3390/ijms242115625 PMC10650169

[6] a) J. Massague , D. Sheppard , Cell 2023, 186, 4007; 37714133 10.1016/j.cell.2023.07.036 PMC10772989

[7] a) S. Colak , P. T. Dijke , Trends in cancer 2017, 3, 56; 28718426 10.1016/j.trecan.2016.11.008

[8] D. Padua , X. H. Zhang , Q. Wang , C. Nadal , W. L. Gerald , R. R. Gomis , J. Massague , Cell 2008, 133, 66. 18394990 10.1016/j.cell.2008.01.046 PMC2390892

[9] X. Tang , L. Shi , N. Xie , Z. Liu , M. Qian , F. Meng , Q. Xu , M. Zhou , X. Cao , W. G. Zhu , B. Liu , Nat. Commun. 2017, 8, 318. 28827661 10.1038/s41467-017-00396-9 PMC5566498

[10] a) W. Wu , X. Wang , X. Yu , H. Y. Lan , Int. J. biological sci. 2022, 18, 2795; 10.7150/ijbs.71595 PMC9066101 35541902

[11] a) M. J. Macias , P. Martin‐Malpartida , J. Massague , Trends Biochem. Sci. 2015, 40, 296; 25935112 10.1016/j.tibs.2015.03.012 PMC4485443

[12] a) M. Petersen , E. Pardali , G. van der Horst , H. Cheung , C. van den Hoogen , G. van der Pluijm , P. Ten Dijke , Oncogene 2010, 29, 1351; 20010874 10.1038/onc.2009.426

[13] R. Oughtred , C. Stark , B. J. Breitkreutz , J. Rust , L. Boucher , C. Chang , N. Kolas , L. O'Donnell , G. Leung , R. McAdam , F. Zhang , S. Dolma , A. Willems , J. Coulombe‐Huntington , A. Chatr‐Aryamontri , K. Dolinski , M. Tyers , Nucleic Acids Res. 2019, 47, D529. 30476227 10.1093/nar/gky1079 PMC6324058

[14] H. Hu , Y. R. Miao , L. H. Jia , Q. Y. Yu , Q. Zhang , A. Y. Guo , Nucleic Acids Res. 2019, 47, D33. 30204897 10.1093/nar/gky822 PMC6323978

[15] a) R. K. Bahia , X. Hao , R. Hassam , O. Cseh , D. A. Bozek , H. A. Luchman , S. Weiss , Nat. Commun. 2023, 14, 5051; 37598220 10.1038/s41467-023-40776-y PMC10439933

[16] B. Gyorffy , Comput. Struct. Biotechnol. J 2021, 19, 4101. 34527184 10.1016/j.csbj.2021.07.014 PMC8339292

[17] a) Y. Zhu , Z. Tao , Y. Chen , S. Lin , M. Zhu , W. Ji , X. Liu , T. Li , X. Hu , Breast Cancer Res. Treat. 2022, 193, 65; 35254603 10.1007/s10549-022-06514-6

[18] a) F. M. Jacobs , D. Greenberg , N. Nguyen , M. Haeussler , A. D. Ewing , S. Katzman , B. Paten , S. R. Salama , D. Haussler , Nature 2014, 516, 242; 25274305 10.1038/nature13760 PMC4268317

[19] a) T. Lyu , Y. Jiang , N. Jia , X. Che , Q. Li , Y. Yu , K. Hua , R. C. Bast Jr. , W. Feng , Int. J. Cancer 2020, 146, 1553; 31503345 10.1002/ijc.32673

[20] C. Fenizia , C. Bottino , S. Corbetta , R. Fittipaldi , P. Floris , G. Gaudenzi , S. Carra , F. Cotelli , G. Vitale , G. Caretti , Nucleic Acids Res. 2019, 47, 1278. 30544196 10.1093/nar/gky1221 PMC6379668

[21] L. Yang , M. Jin , K. W. Jeong , Biology 2021, 10, 581. 34201935 10.3390/biology10070581 PMC8301125

[22] A. Peserico , A. Germani , P. Sanese , A. J. Barbosa , V. Di Virgilio , R. Fittipaldi , E. Fabini , C. Bertucci , G. Varchi , M. P. Moyer , G. Caretti , A. Del Rio , C. Simone , J. Cell. Physiol. 2015, 230, 2447. 25728514 10.1002/jcp.24975 PMC4988495

[23] S. K. Wculek , I. Malanchi , Nature 2015, 528, 413. 26649828 10.1038/nature16140 PMC4700594

[24] J. Jin , L. Bai , D. Wang , W. Ding , Z. Cao , P. Yan , Y. Li , L. Xi , Y. Wang , X. Zheng , H. Wei , C. Ding , EMBO Rep. 2023, 24, e56052. 36896611 10.15252/embr.202256052 PMC10157311

[25] J. Jiang , D. Huang , Y. Jiang , J. Hou , M. Tian , J. Li , L. Sun , Y. Zhang , T. Zhang , Z. Li , S. Tong , Y. Ma , Frontiers in oncology 2021, 11, 647559. 34150616 10.3389/fonc.2021.647559 PMC8208031

[26] a) S. Hong , H. J. Lee , S. J. Kim , K. B. Hahm , World journal of gastroenterology. 2010, 16, 2080; 20440848 10.3748/wjg.v16.i17.2080 PMC2864833

[27] a) J. Massague , J. Seoane , D. Wotton , Genes Dev. 2005, 19, 2783; 16322555 10.1101/gad.1350705

[28] A. Bertero , S. Brown , P. Madrigal , A. Osnato , D. Ortmann , L. Yiangou , J. Kadiwala , N. C. Hubner , I. R. de Los Mozos , C. Sadee , A. S. Lenaerts , S. Nakanoh , R. Grandy , E. Farnell , J. Ule , H. G. Stunnenberg , S. Mendjan , L. Vallier , Nature 2018, 555, 256. 29489750 10.1038/nature25784 PMC5951268

[29] a) T. Rogers , R. J. DeBerardinis , Trends in cancer 2021, 7, 700; 34023325 10.1016/j.trecan.2021.04.007 PMC9270875

[30] a) E. Nolan , V. L. Bridgeman , L. Ombrato , A. Karoutas , N. Rabas , C. A. N. Sewnath , M. Vasquez , F. S. Rodrigues , S. Horswell , P. Faull , R. Carter , I. Malanchi , Nature cancer 2022, 3, 173; 35221334 10.1038/s43018-022-00336-7 PMC7612918

[31] O. Rosspopoff , D. Trono , Trends in genetics : TIG. 2023, 39, 844. 37716846 10.1016/j.tig.2023.08.003

[32] a) P. Y. Helleboid , M. Heusel , J. Duc , C. Piot , C. W. Thorball , A. Coluccio , J. Pontis , M. Imbeault , P. Turelli , R. Aebersold , D. Trono , EMBO J. 2019, 38, 101220; 10.15252/embj.2018101220 PMC6745500 31403225

[33] a) I. Barde , B. Rauwel , R. M. Marin‐Florez , A. Corsinotti , E. Laurenti , S. Verp , S. Offner , J. Marquis , A. Kapopoulou , J. Vanicek , D. Trono , Science 2013, 340, 350; 23493425 10.1126/science.1232398 PMC3678075

[34] X. Zhang , Q. Zheng , X. Yue , Z. Yuan , J. Ling , Y. Yuan , Y. Liang , A. Sun , Y. Liu , H. Li , K. Xu , F. He , J. Wang , J. Wu , C. Zhao , C. Tian , J. exp. & Clin. Canc. Res.: CR. 2022, 41,79. 10.1186/s13046-022-02288-3 PMC8883630 35227287

[35] a) Z. A. Gibbs , L. C. Reza , C. C. Cheng , J. M. Westcott , K. McGlynn , A. W. Whitehurst , eLife 2020, 9; 10.7554/eLife.57679 PMC7302877 32515734

[36] a) C. Li , L. Cao , C. Xu , F. Liu , G. Xiang , X. Liu , J. Jiao , Y. Niu , Human pathology. 2018, 75, 16; 29180246 10.1016/j.humpath.2017.11.010

[37] Z. Wang , M. Wang , M. Zhang , K. Xu , X. Zhang , Y. Xie , Y. Zhang , C. Chang , X. Li , A. Sun , F. He , BMC medicine. 2022, 20, 292. 35941608 10.1186/s12916-022-02436-8 PMC9361549

[38] J. Wang , X. Zhang , J. Ling , Y. Wang , X. Xu , Y. Liu , C. Jin , J. Ju , Y. Yuan , F. He , C. Zhao , C. Tian , Biochimica et biophysica acta. Gene regulatory mechanisms. 2018, S1874, 30048. 10.1016/j.bbagrm.2018.07.003 30012466

