# Supplementary material for: ZNF8 Orchestrates with Smad3 to Promote Lung Metastasis by Recruiting SMYD3 in Breast Cancer
Source: Adv Sci (Weinh). 2024 Sep 3;11(40):2404904. doi: 10.1002/advs.202404904 (PMC11515916; doi:10.1002/advs.202404904)
Supplement: Supplementary file 1 — Supporting Information [file ADVS-11-2404904-s002.docx]

Supporting Information

**Title：ZNF8 orchestrates with Smad3 to promote lung metastasis by recruiting SMYD3 in breast cancer**

*Wenwen Geng^1,2^, Junhua An^1,2^, Ke Dong^1,2^, Hailu Zhang^3^, Xiuyuan Zhang^3^, Yuchen Liu^3^, Rong Xu^3^, Yifan Liu^3^, Xiaofen Huang^4^, Haiyun Song^5^, Wei Yan^6^, Aihua Sun^3,4,7^, Fuchu He^3,7^, Jian Wang^3,4*^, Haidong Gao^1, 2 *^, Chunyan Tian^3,4,7*^*

^1^Department of Breast Surgery, Qilu Hospital (Qingdao), Cheeloo College of Medicine, Shandong University, Qingdao 266000, Shandong, China;

^2^Laboratory of Oncology, Qilu Hospital (Qingdao), Cheeloo College of Medicine, Shandong University, Qingdao 266000, Shandong, China；

^3^State Key Laboratory of Medical Proteomics, Beijing Proteome Research Center, National Center for Protein Sciences (Beijing), Beijing Institute of Lifeomics, Beijing 102206, China;

^4^College of Life Sciences, Hebei University, Baoding 071002, Hebei, China;

^5^Department of Pathology, Qilu Hospital (Qingdao), Cheeloo College of Medicine, Shandong University, Qingdao 266000, Shandong, China;

^6^The First Medical Center of Chinese PLA General Hospital, Beijing 100036, China;

^7^Research Unit of Proteomics Dirven Cancer Precision Medicine, Chinese Academy of Medical Sciences, Beijing 102206, China;

^*^Corresponding author

**Supplementary Figures**


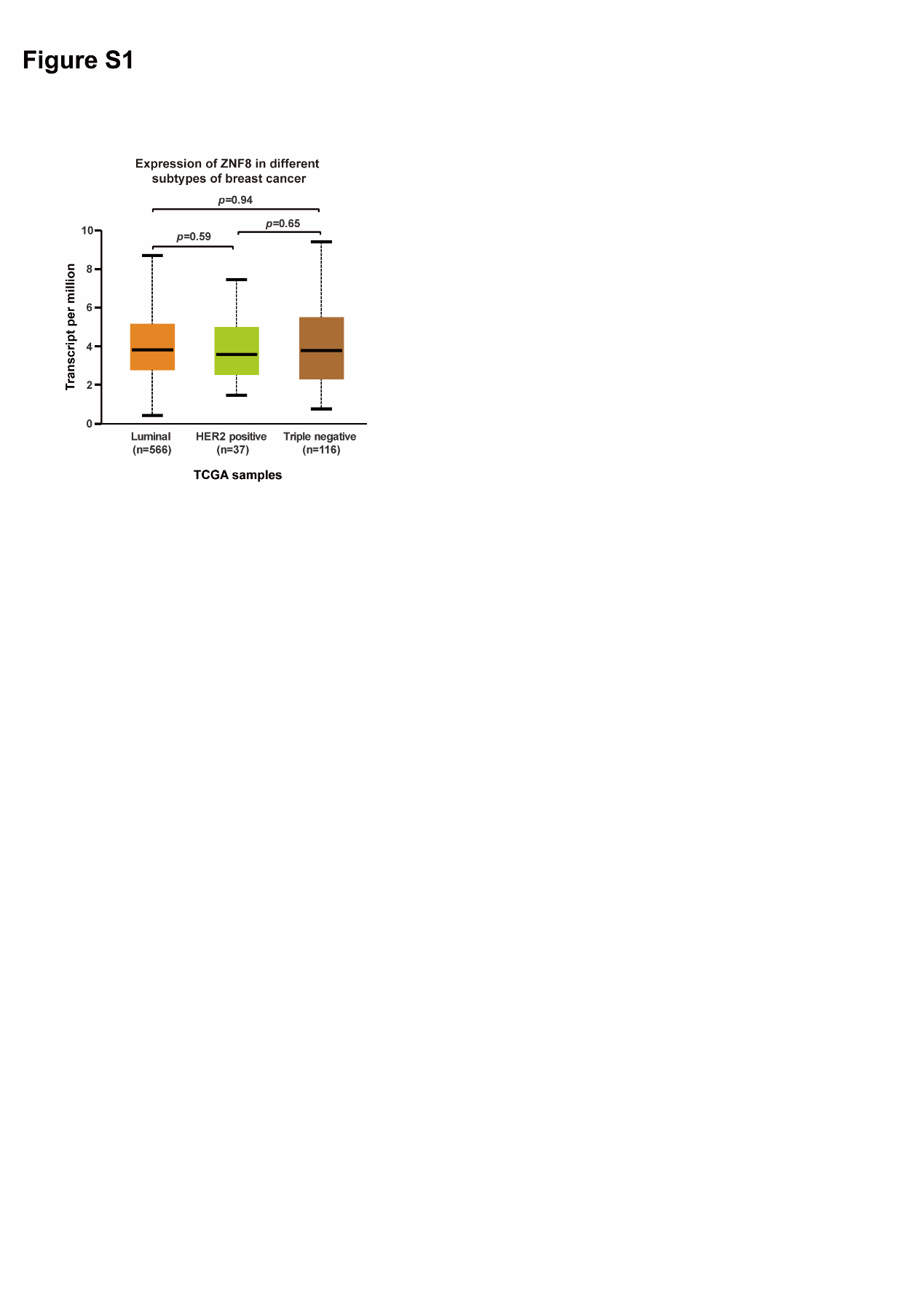


**Figure S1. Expression of ZNF8 in different subtypes of breast cancer in TCGA database.**


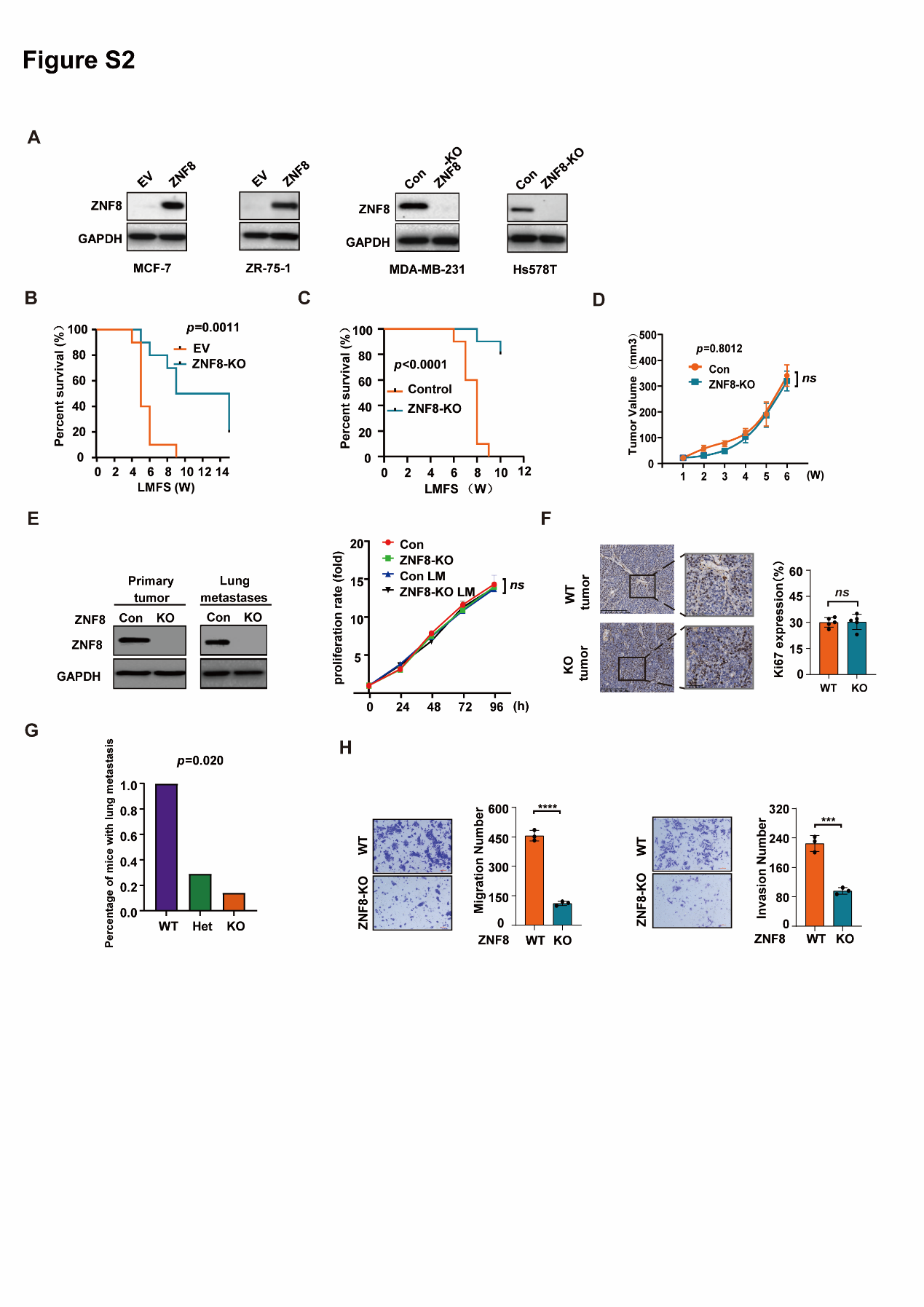


**Figure S2. ZNF8 promotes breast cancer lung metastasis**

**(A)** Representative Western blots for ZNF8 and the loading control GAPDH in ZNF8 overexpressing ZR-75-1 and MCF7 cells, and in ZNF8 knockout MDA-MB-231 and Hs578T cells.

**(B)** Incidence of lung metastasis in tail vein injection metastasis model with ZNF8 knockout and control MDA-MB-231 cells (n=6 mice/group).

**(C)** Incidence of lung metastasis of orthotopic ZNF8 knockout and control MDA-MB-231 cells (n=10 mice/group).

**(D)** The plot of the growth of tumors in situ after orthotopic implantation by mammary fat pad injections.

**(E)** Identification the tumor cells isolated from orthotopic and lung metastatic tumors (left panel), and analysis of the cell proliferation (right panel) (n≥3).

**(F) Representative Immunohistochemical (IHC) staining and analysis of Ki67 expression in breast tumors of WT and KO mice.**

**(G)** Percentage of KO, Het, and WT animals (n=7 mice/group) mice with lung metastasis 23 weeks after birth.

**(H) Representative images and quantification of Transwell assay for migration and invasion in tumor cells isolated from lung metastasis lesion of WT and KO mice (n≥3).** For B and C, significance was determined with Log–rank (Mantel–Cox) test. For D, F,H significance was determined with unpaired t test. For E, significance was determined with One-way ANOVA**.**


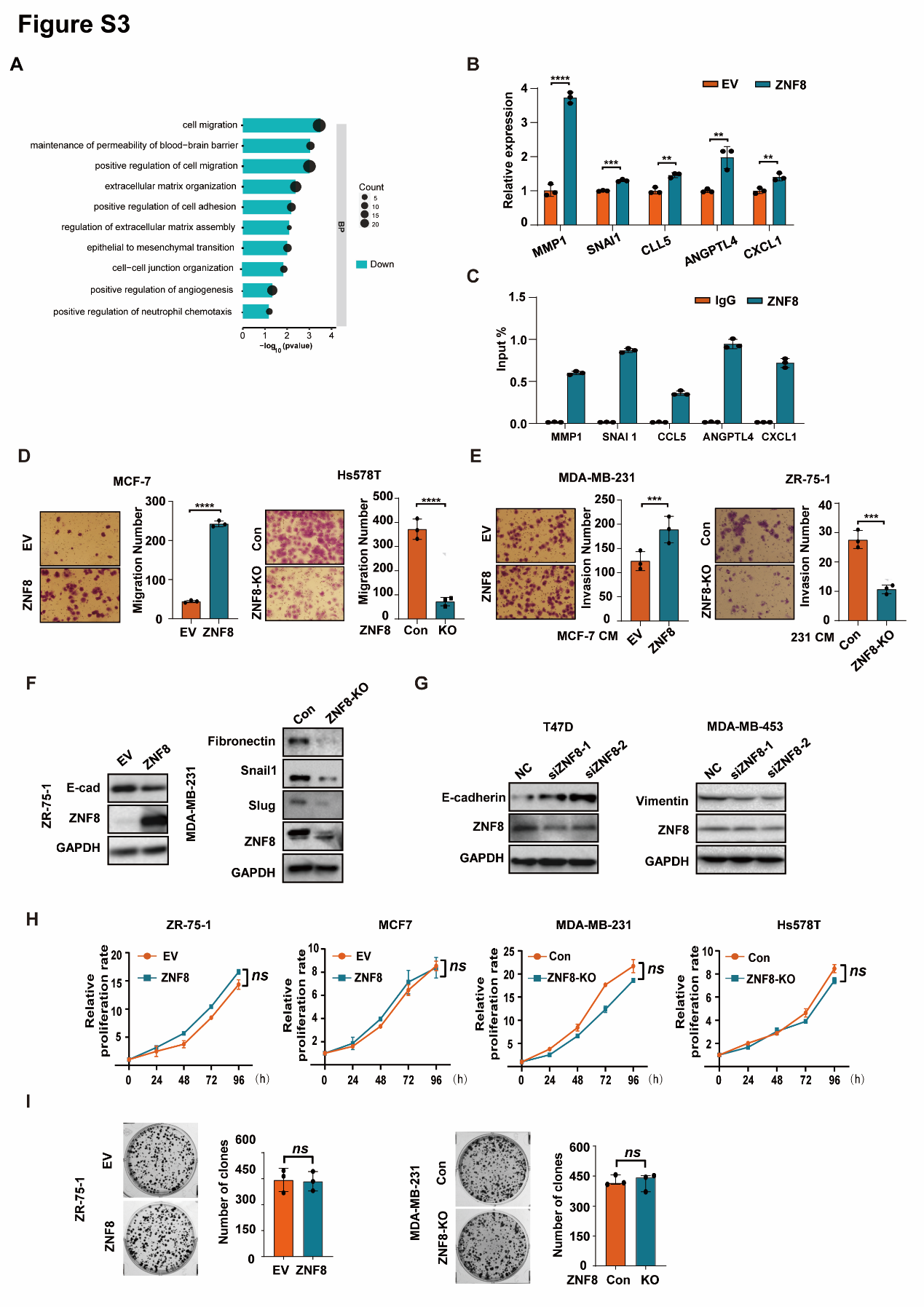


**Figure S3. ZNF8 is required for the multiple processes in the metastatic cascades**

**(A)** Gene-ontology analysis (BP) of the downregulation genes in ZNF8 knockout MDA-MB-231 cells (*p-*adj <0.05).

(B) RT–qPCR analysis of signature genes MMP1, SNAI1, CCL5, ANGPTL4 and CXCL1 in ZNF8 overexpression MCF-7 cells.

**(C)** ChIP-qPCR for ZNF8 occupancy at signature genes promoter in MDA-MB-231 cells as quantified by % of Input.

**(D)** Representative images and quantification of Transwell assay for migration in MCF-7 cells (empty vector vs.ZNF8 overexpression) (left panel), and in Hs578T cells (control vs.ZNF8 knockout) (fight panel) (n≥3).

**(E)** Representative images and quantification of Transwell assay for invasion in MDA-MB-231 cells treated with conditioned medium form ZNF8 overexpression MCF-7 cells and ZNF8 knockout MDA-MB-231 cells, and in ZR-75-1 cells treated with conditioned medium form ZNF8 knockout MDA-MB-231 cells (n≥3).

**(F)** Representative Western blots for E-cadherin, ZNF8 and the loading control GAPDH in ZR-75-1, and for Fibronectin, Snail, Slug, ZNF8 and the loading control GAPDH in MDA-MB-231 cells (n≥3).

**(G)** Representative Western blots for E-cadherin, ZNF8 and the loading control GAPDH in T47D cells with ZNF8 knock down by siRNA, and for Vimentin, ZNF8 and the loading control GAPDH in MDA-MB-231 cells with ZNF8 knock down by siRNA (n≥3).

**(H)** The plot of the growth in empty vector and ZNF8 overexpression ZR-75-1 and MCF-7 cells, and in control and ZNF8 knockout MDA-MB-231 and Hs578T cells (n≥3).

**(F)** Representative images and quantification of colony formation assay in ZR-75-1 cells (empty vector vs. ZNF8 overexpression) and MDA-MB-231 cells (control vs. ZNF8 knockout) (n≥3).

For B-E, H and I, data represent mean ± SD, and significance was determined with the student unpaired t test. ns, *p* > 0.05; *, *p*< 0.05; **, *p* < 0.01; ***, *p* < 0.001; ****, *p*< 0.0001.


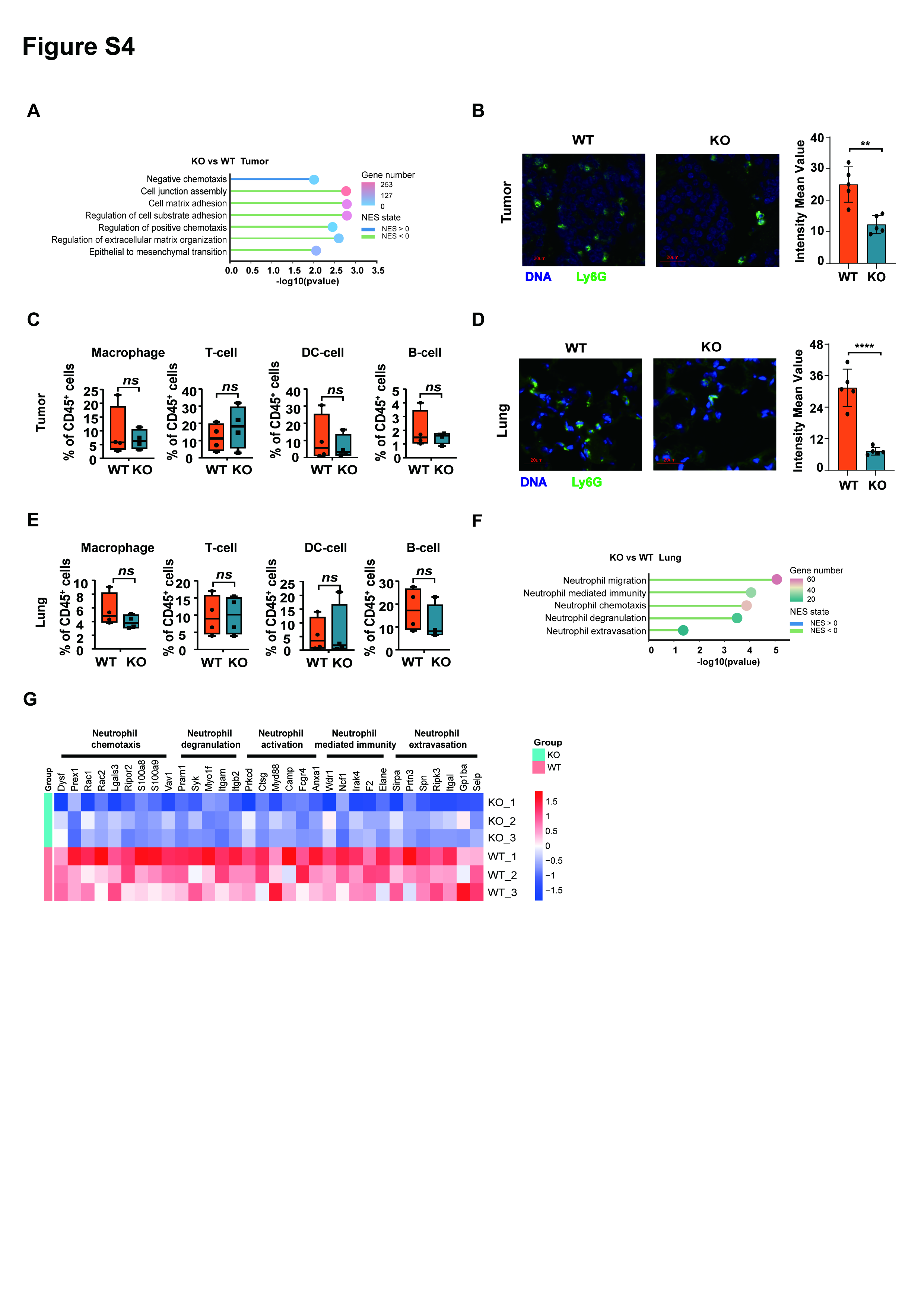


**Figure S4. ZNF8 promotes neutrophil infiltration in the primary tumor and lung tissues.**

**(A, F)** GSEA plots by normalized enrichment score (NES) of pathways that correlate significantly with ZNF8 protein expression in tumors (A) and lung (F) of transgenic mice 20 week from birth (green: enriched with WT, blue: enriched with ZNF8 knockout; nominal *p* values are indicated, n=5).

**(B)** Representative confocal images of neutrophil (Ly6G+) in tumors of WT and KO mice.

**(C)** Quantification of infiltration for macrophage cell, T-cell, DC-cell and B-cell in tumors from transgenic mice 20week from birth (n=5).

**(D)** Representative confocal images of neutrophil (Ly6G+) in lungs of WT and KO mice

**(E)** Quantification of infiltration for macrophage cell, T-cell, DC-cell and B-cell in lungs from transgenic mice 20week from birth (n=5).

**(G)** Heatmap from proteomic analysis of the proteins associated with neutrophil chemotaxis, neutrophil degranulation, neutrophil activation, neutrophil mediated immunity and neutrophil extravasation in lungs of transgenic mice 20week from birth (WT vs.ZNF8 knockout) (*p-*adj <0.05). For C and D, data represent mean ± SD, and significance was determined with the student unpaired t test. *ns*.


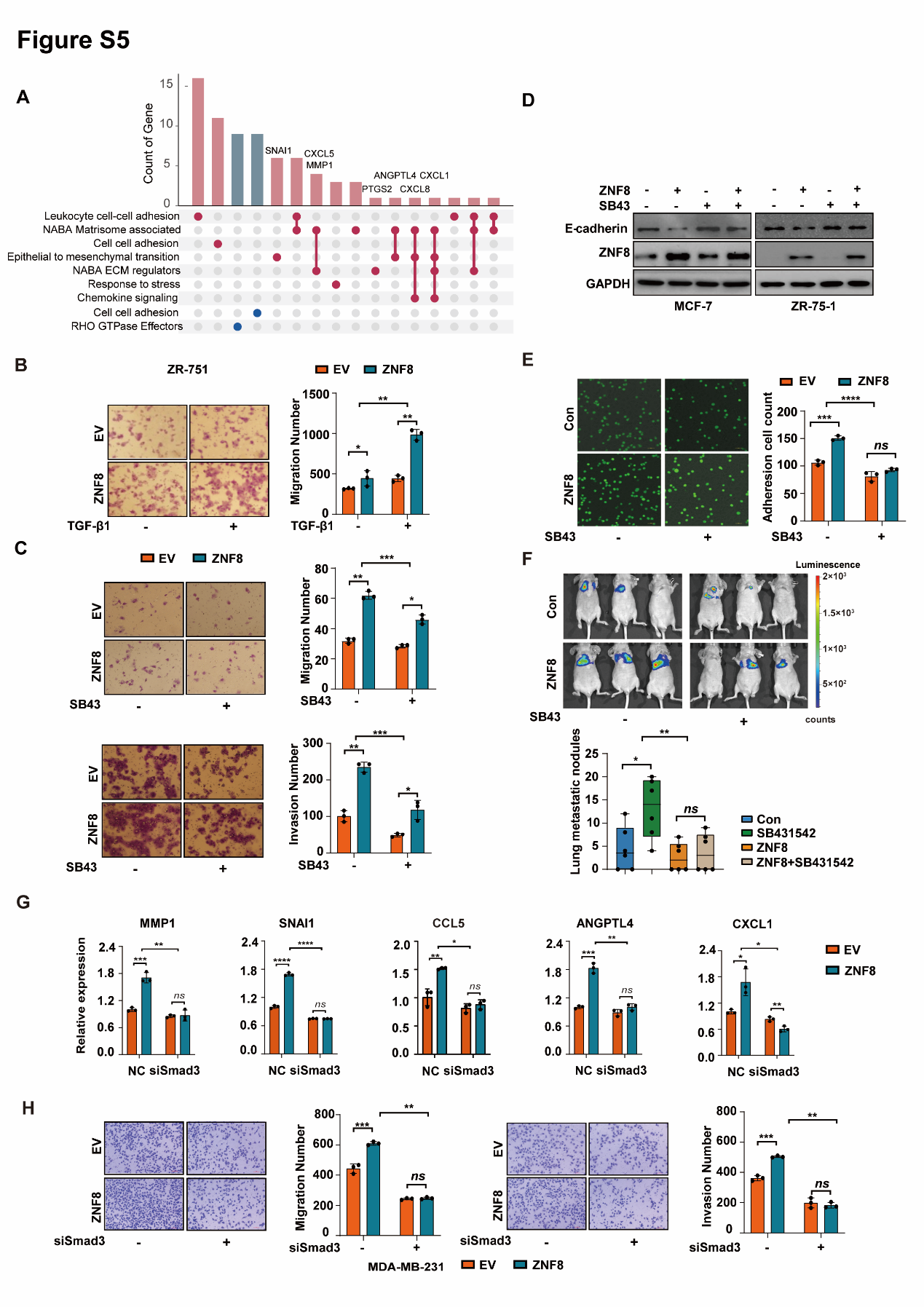


**Figure S5. ZNF8 promotes metastasis in dependence of TGF-β/Smad3 pathway**

**(A)** Gene-ontology analysis of the overlap genes that regulated by ZNF8 knockout as well as TGF-β pathway inhibitor SB431542 treatment in MDA-MB-231 cells.

**(B)** Representative images and quantification of Transwell assay for migration in ZNF8 overexpression ZR-75-1 cells with TGF-β1 treatment (n≥3).

**(C)** Representative images and quantification of Transwell assay for migration and invasion in ZNF8 overexpression ZR-75-1 cells with SB431542 treatment (n≥3).

**(D)** Representative Western blots for E-cadherin, ZNF8 and the loading control GAPDH in ZNF8 overexpression MCF7 and ZR-75-1 cells with SB431542 treatment (n≥3).

**(E)** Representative images and quantification of cell adhesion assay in ZNF8 overexpression MDA-MB-231 cells with SB431542 treatment. Cell number was counted in six randomly captured pictures (n≥3). Scale bar, 50 µm.

**(F)** Representative images of vivo bioluminescence imaging for the study of lung metastasis via tail-vein injection of empty vector and ZNF8 overexpression MDA-MB-231 breast cancer cells treated with SB431542 (upper panel). Quantification of lung metastatic nodules in lung tissues harvested at Week15 (down panel) (n=6 mice/group).

**(G)** RT–qPCR analysis of signature genes MMP1, SNAI1, CCL5, ANGPTL4 and CXCL1 in ZNF8 overexpression MDA-MB-231 cells with Smad3 knockdown (n≥3).

**(H)** Representative images and quantification of Transwell assay for migration and invasion in ZNF8 overexpression MDA-MB-231 cells with Smad3 knockdown (n≥3). For B C, E, F, G and H, data represent mean ± SD, and significance was determined with the student unpaired t test and Two-way ANOVA. *ns*, *p* > 0.05; *, *p*< 0.05; **, *p* < 0.01; ***, *p* < 0.001; ****, *p*< 0.0001.

.


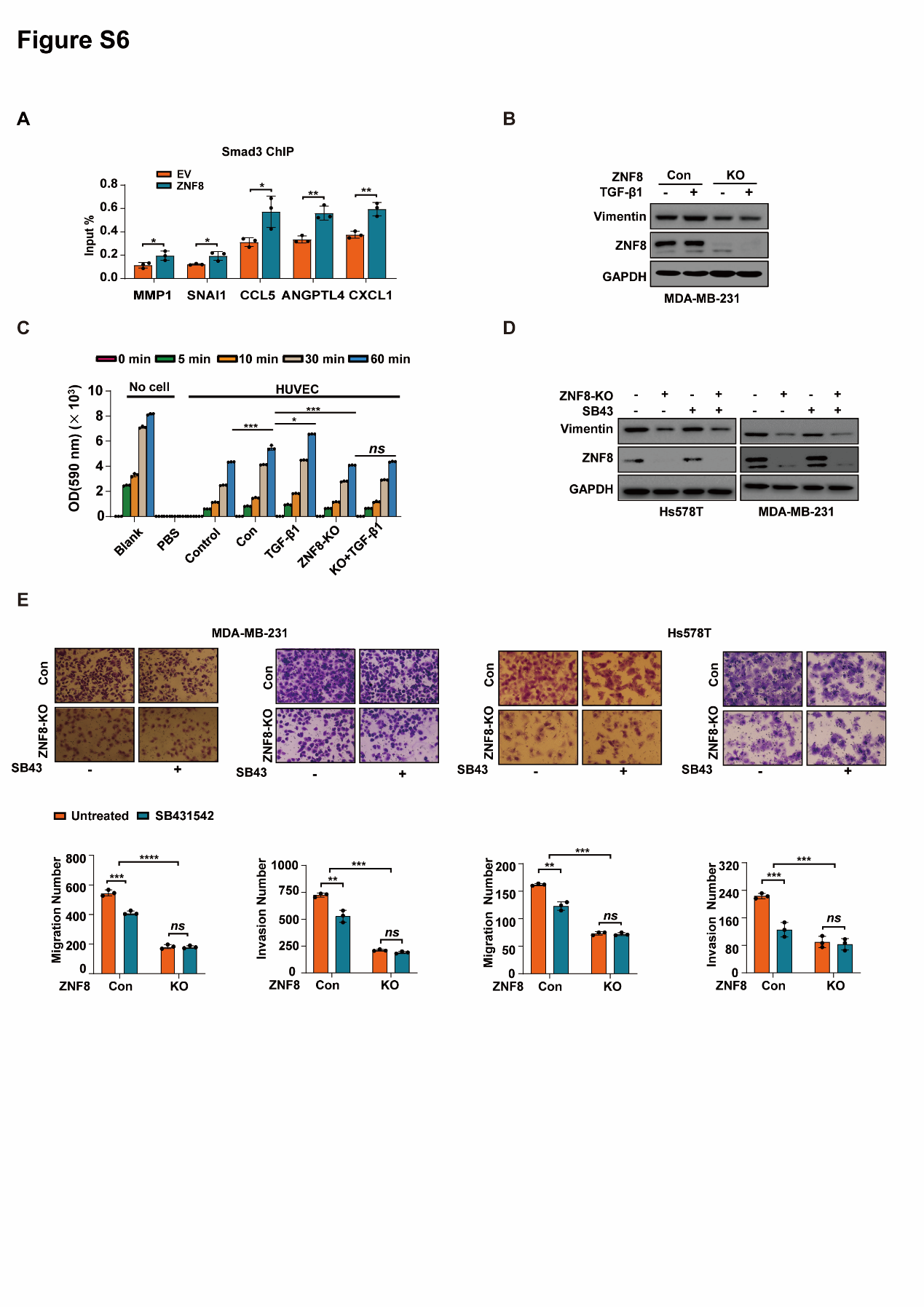


**Figure S6. ZNF8 is indispensable for the TGF-β signaling pathway-mediated metastasis in breast cancer cells**

**(A)** ChIP-qPCR for Smad3 occupancy at signature genes promoter in empty vector and ZNF8 overexpression MDA-MB-231 cells as quantified by % of Input.

**(B)** Representative Western blots for Vimentin, ZNF8 and the loading control GAPDH in control and ZNF8 knockout MDA-MB-231 and Hs578T cells with TGF-β1 treatment.

**(C)** Quantification of endothelial permeability in control and ZNF8 knockout MDA-MB-231 cells with TGF-β1 treatment (n≥3).

**(D)** Representative Western blots for Vimentin, ZNF8 and the loading control GAPDH in control and ZNF8 knockout MDA-MB-231 cells with SB431542 treatment.

**(E)** Representative images and quantification of Transwell assay for migration and invasion in control and ZNF8 knockout MDA-MB-231 and Hs578T cells with SB431542 treatment (n≥3). For A and C, data represent mean ± SD, and significance was determined with the Student's t test and Two-way ANOVA, *ns*, *p* > 0.05; *, *p*< 0.05; **, *p* < 0.01; ***, *p* < 0.001; ****, *p*< 0.0001.


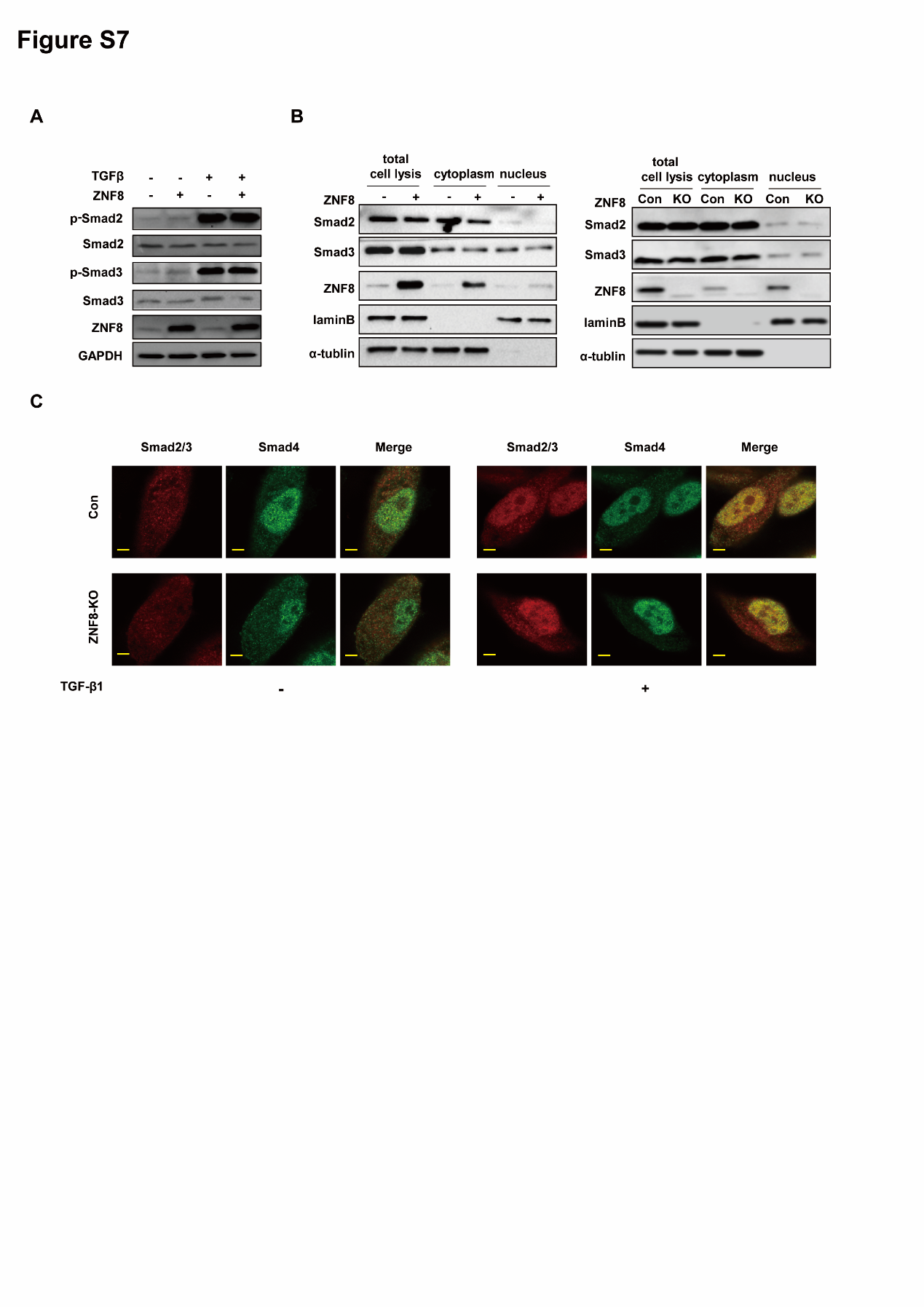


**Figure S7. ZNF8 is unnecessary for Smad2/3 phosphorylation and nuclear translocation**

**(A)** Representative Western blots for p-Smad2, Smad2, p-Smad3, Smad3, ZNF8 and the loading control GAPDH in control and ZNF8 knockout MDA-MB-231 cells with TGF-β1 treatment (n≥3).

**(B)** Representative Western blots for Smad2, Smad3, ZNF8, the loading control laminB and α-tublin in empty vector and ZNF8-overexpressing, and in control and ZNF8 knockout MDA-MB-231 cells (n≥3).

**(C)** Representative confocal images of Smad2/3 nuclear translocation and colocation with Smad4 in control and ZNF8 knockout MDA-MB-231 cells with TGF-β1 treatment. bar=5μm.


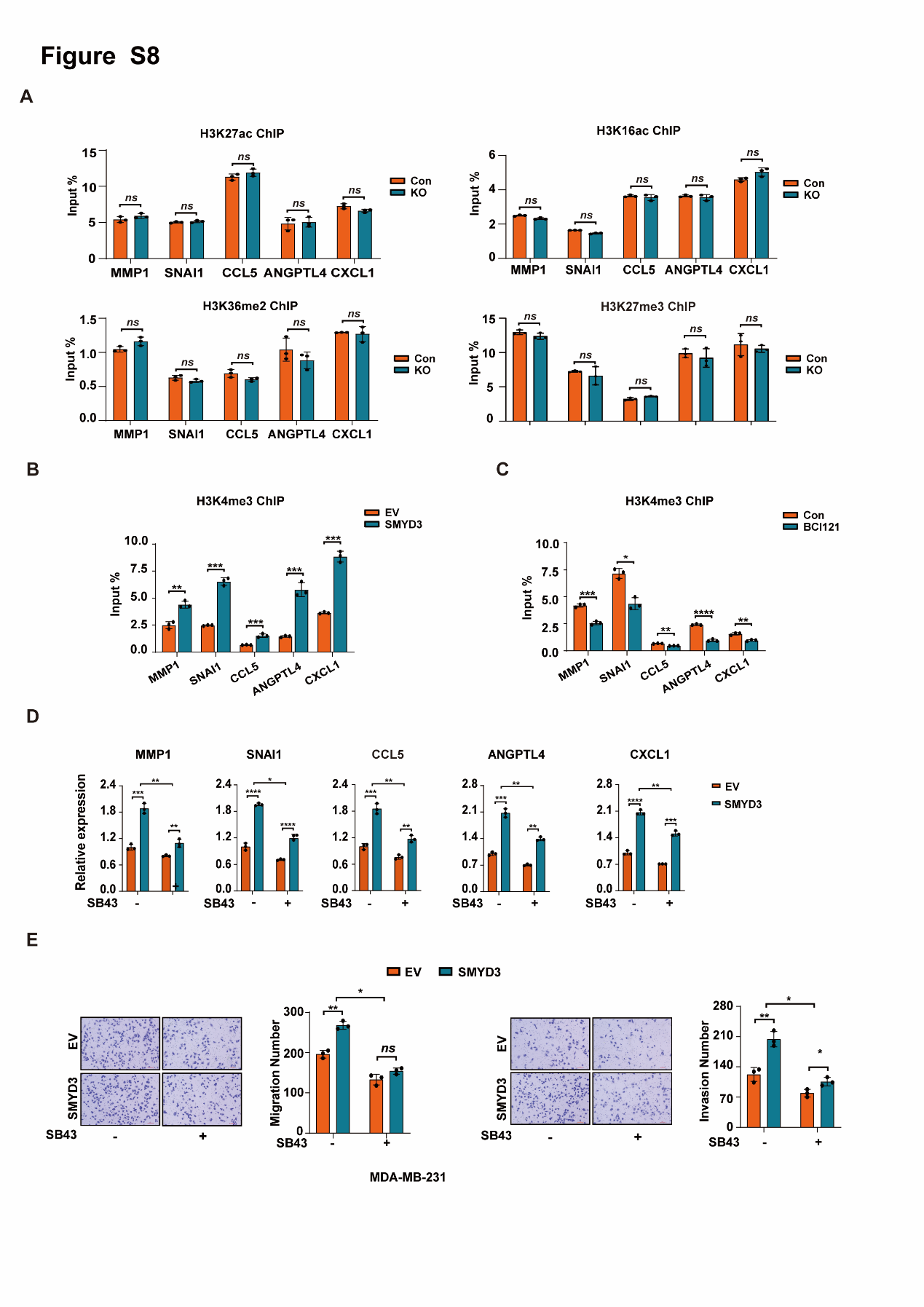


**Figure S8. SMYD3 increases the H3K4me3 level and expression of lung metastasis signature genes, and enhances the migration and invasion abilities through TGF-β pathway.**

**(A)** ChIP-qPCR for H3K27ac, H3K16ac, H3k36me2 and H3K27me3 occupancy at promoter of lung metastasis signature genes in ZNF8 knockout and overexpressing MDA-MB-231 cells as quantified by % of Input (n≥3).

**(B)** ChIP-qPCR for H3K4me3 occupancy at promoter of lung metastasis signature genes in SMYD3 overexpression MDA-MB-231 cells as quantified by % of Input (n≥3).

**(C)** ChIP-qPCR for H3K4me3 occupancy at promoter of lung metastasis signature genes in MDA-MB-231 cells treated with BCI121.

**(D)** RT–qPCR analysis of signature genes MMP1, SNAI1, CCL5, ANGPTL4 and CXCL1 in SMYD3 overexpression MDA-MB-231 cells with SB431542 treatment (n≥3).

**(E)** Representative images and quantification of Transwell assay for migration and invasion in SMYD3 overexpression MDA-MB-231 cells with SB431542 treatment (n≥3).

For A-C, data represent mean ± SD, and significance was determined with the Student's t test and Two-way ANOVA; For D and E, data represent mean ± SD, and significance was determined with the Student's t test and Two-way ANOVA *ns*, *p* > 0.05; *, *p*< 0.05; **, *p* < 0.01; ***, *p* < 0.001; ****, *p*< 0.0001.

**
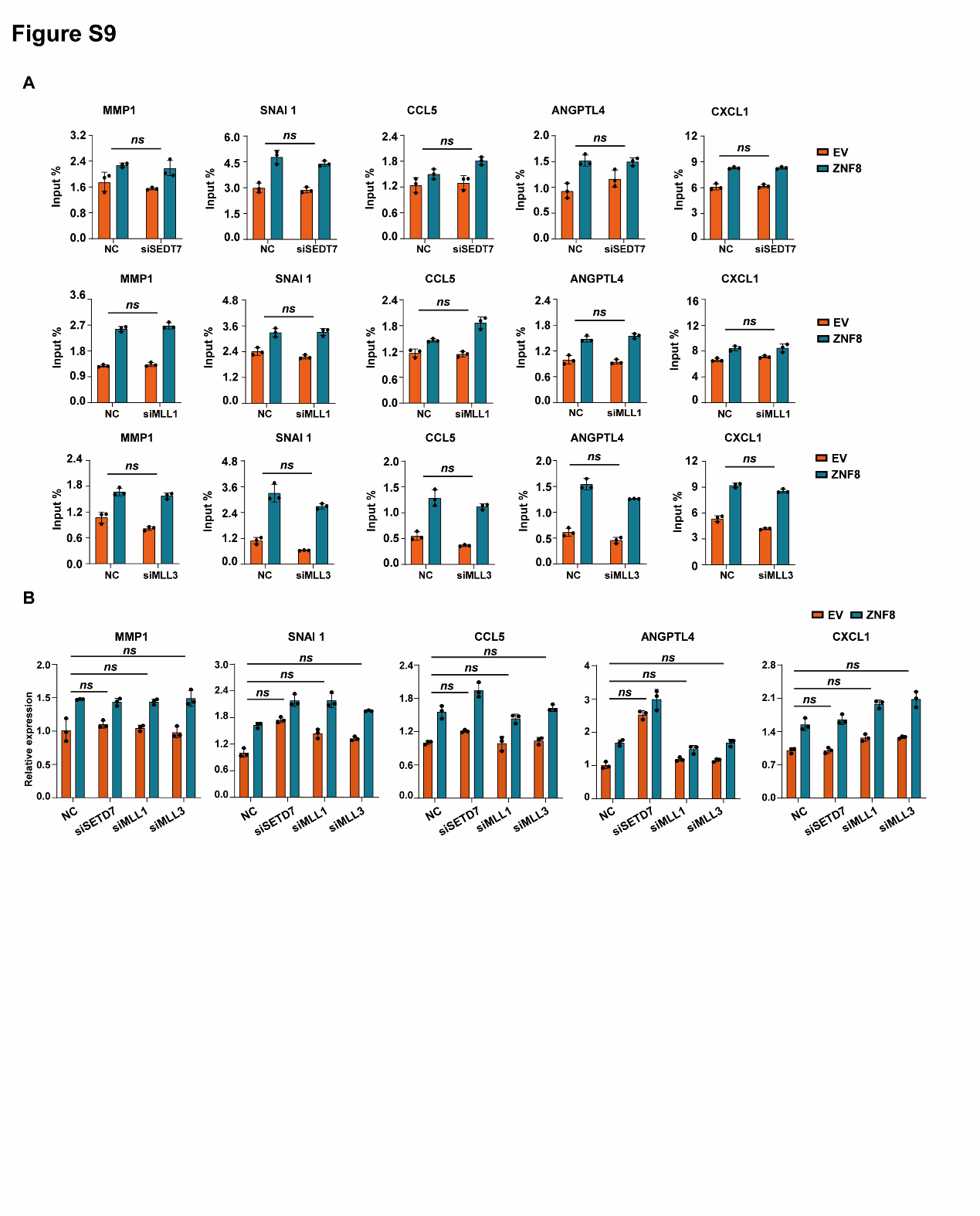
**

**Figure S9. The function of ZNF8 was not depending on H3K4 methyltransferases SETD7, MLL1, MLL3.**

**(A)** ChIP-qPCR for H3K4me3 occupancy at promoter of lung metastasis signature genes in empty vector and ZNF8 overexpression MDA-MB-231 cells with knockdown of SETD7, MLL1 and MLL3 by siRNA as quantified by % of Input (n≥3).

**(B)** RT–qPCR analysis of signature genes MMP1, SNAI1, CCL5, ANGPTL4 and CXCL1 in ZNF8 overexpression MDA-MB-231 cells with knockdown of SETD7, MLL1 and MLL3 by siRNA (n≥3). For A-C, data represent mean ± SD, and significance was determined with the Student's t test and Two-way ANOVA; For D and E, data represent mean ± SD, and significance was determined with Two-way ANOVA *ns*, *p* > 0.05.

**
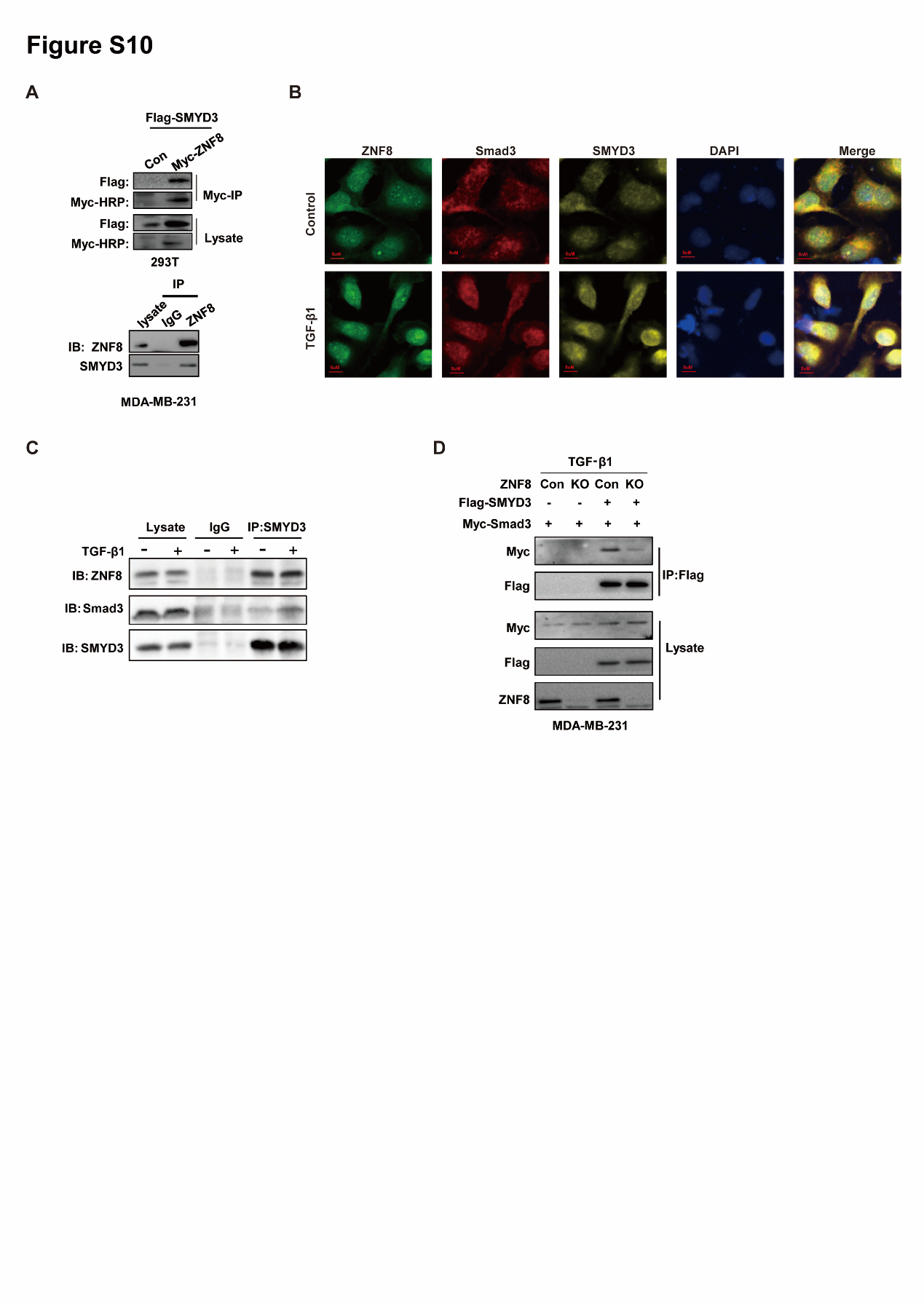
**

**Figure S10. ZNF8 interacts with SMYD3 and recruits it to Smad3**

**(A)** Co-immunoprecipitation with anti-Myc antibody in HEK293T cells co-transfected with Myc-ZNF8 and Flag-SMYD3 (left panel). Co-immunoprecipitation of endogenous SMYD3 with anti ZNF8 antibodies in MDA-MB-231 cells (right panel).

(B) Representative confocal images for the nuclear colocalization of Smad3, ZNF8 and SMYD3 in MDA-MB-231 cells with TGF-β1 treatment. bar=5 μm.

**(C)** Co-immunoprecipitation of endogenous Smad3, ZNF8 using SMYD3 antibodies in MDA-MB-231 breast cancer cells.

**(D)** Co-immunoprecipitation with anti-Flag antibody in MDA-MB-231 cells co-transfected with Myc-Smad3 and Flag-SMYD3 with TGF-β1 treatment.


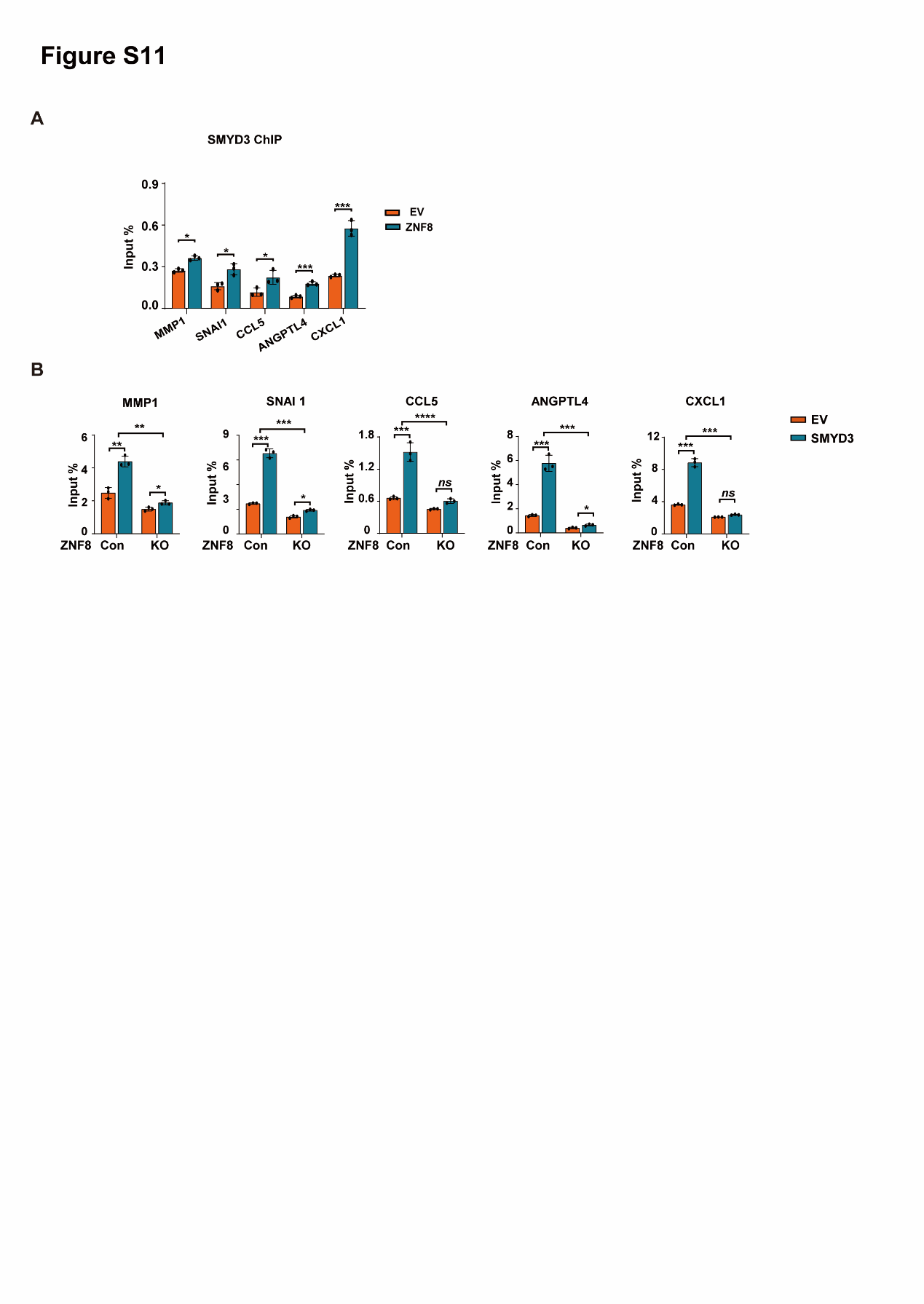


**Figure S11. The promoting effect of SMYD3 on the signature genes is regulated by ZNF8**

**(A)** ChIP-qPCR for SMYD3 occupancy at signature genes promoter in empty vector and ZNF8 overexpression MDA-MB-231 cells as quantified by % of Input.

**(B)** ChIP-qPCR for H3K4me3 occupancy at promoter of lung metastasis signature genes in ZNF8 knockout MDA-MB-231 cells with SMYD3 overexpression as quantified by % of Input (n≥3). For D and G, data represent mean ± SD, and significance was determined with the Student's t test, *ns*, *p* > 0.05; *, *p*< 0.05; **, *p* < 0.01; ***, *p* < 0.001; ****, *p*< 0.0001.


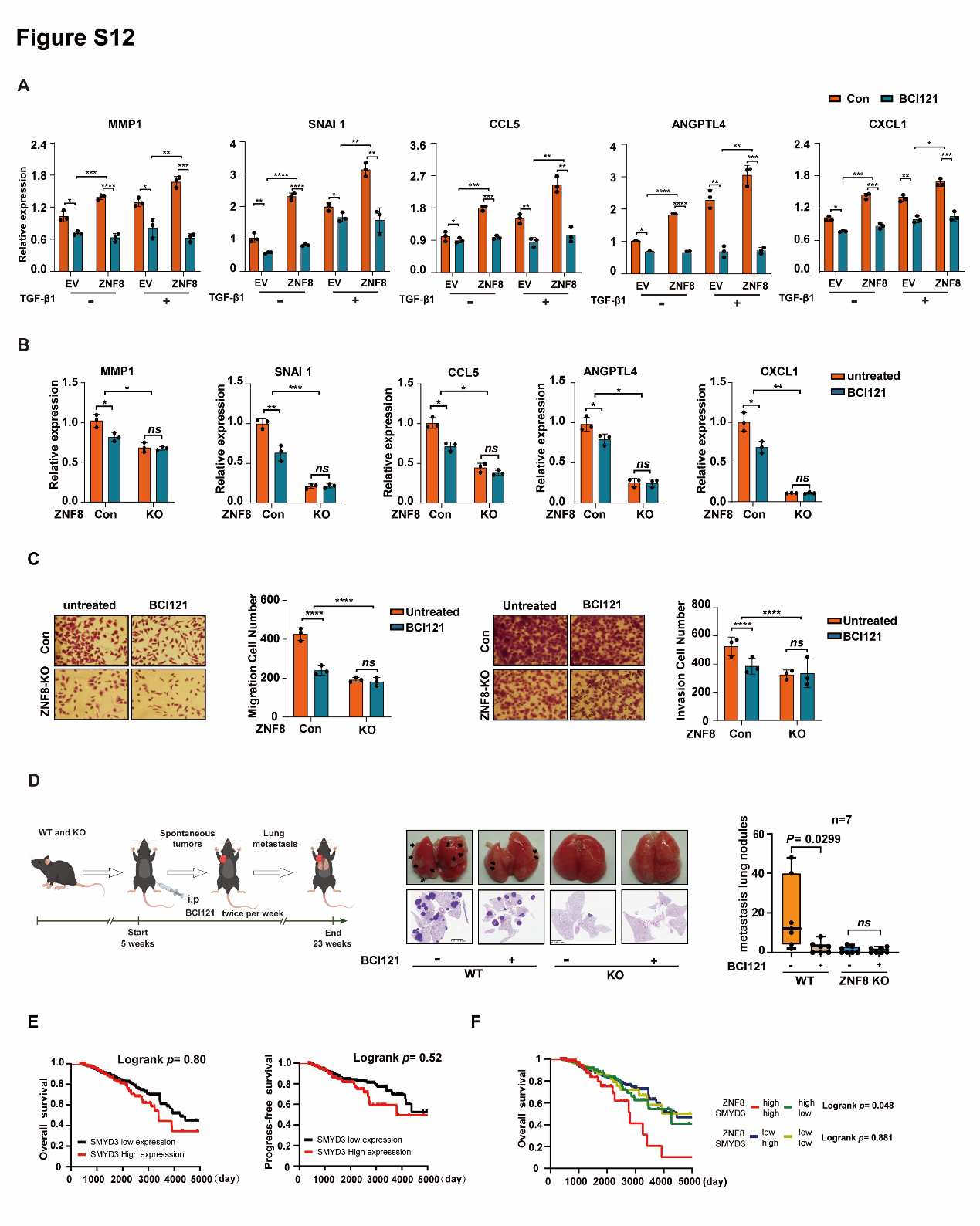


**Figure S12. ZNF8 KO abolishes the sensitivity to SMYD3 inhibition in breast cancer**

**(A)** RT–qPCR analysis of lung metastasis signature genes in control and ZNF8 knockout MDA-MB-231 cells with BCI121 treatment (n≥3).

**(B)** RT–qPCR analysis of lung metastasis signature genes in control and ZNF8 knockout MDA-MB-231 cells with BCI121 treatment (n≥3).

**(C)** Representative images and quantification of Transwell assay for migration and invasion in ZNF8 knockout MDA-MB-231 cells with BCI121 treatment (n≥3).

**(D)** Schematic diagram of the study of transgenic mice treated with BCI121, (n=7 mice/group) (left panel). Representative images and quantification of lung metastatic nodules in lung tissues harvested at Week 23 after birth, (n=7 mice/group), (right panel).

**(E)** Kaplan-Meier overall survival (n=911) (left panel), and progress-free survival (n=885) (right panel) stratified by low SMDY3 (black) and high SMDY3 (red) mRNA expression in TCGA Breast cancer cases.

**(F)** Kaplan-Meier overall survival stratified by high ZNF8 and SMDY3 (red), high ZNF8 and low SMYD3 (green), low ZNF8 and high SMDY3 (blue), low ZNF8 and SMYD3 (yellow) mRNA expression in TCGA Breast cancer cases, (n=911). For A-C, data represent mean ± SD, and significance was determined with the Student's t test, ns, *p* > 0.05; *, *p*< 0.05; **, *p* < 0.01; ***, *p* < 0.001; ****, *p*< 0.0001. For D and E, significance was determined with Log–rank (Mantel–Cox) test.


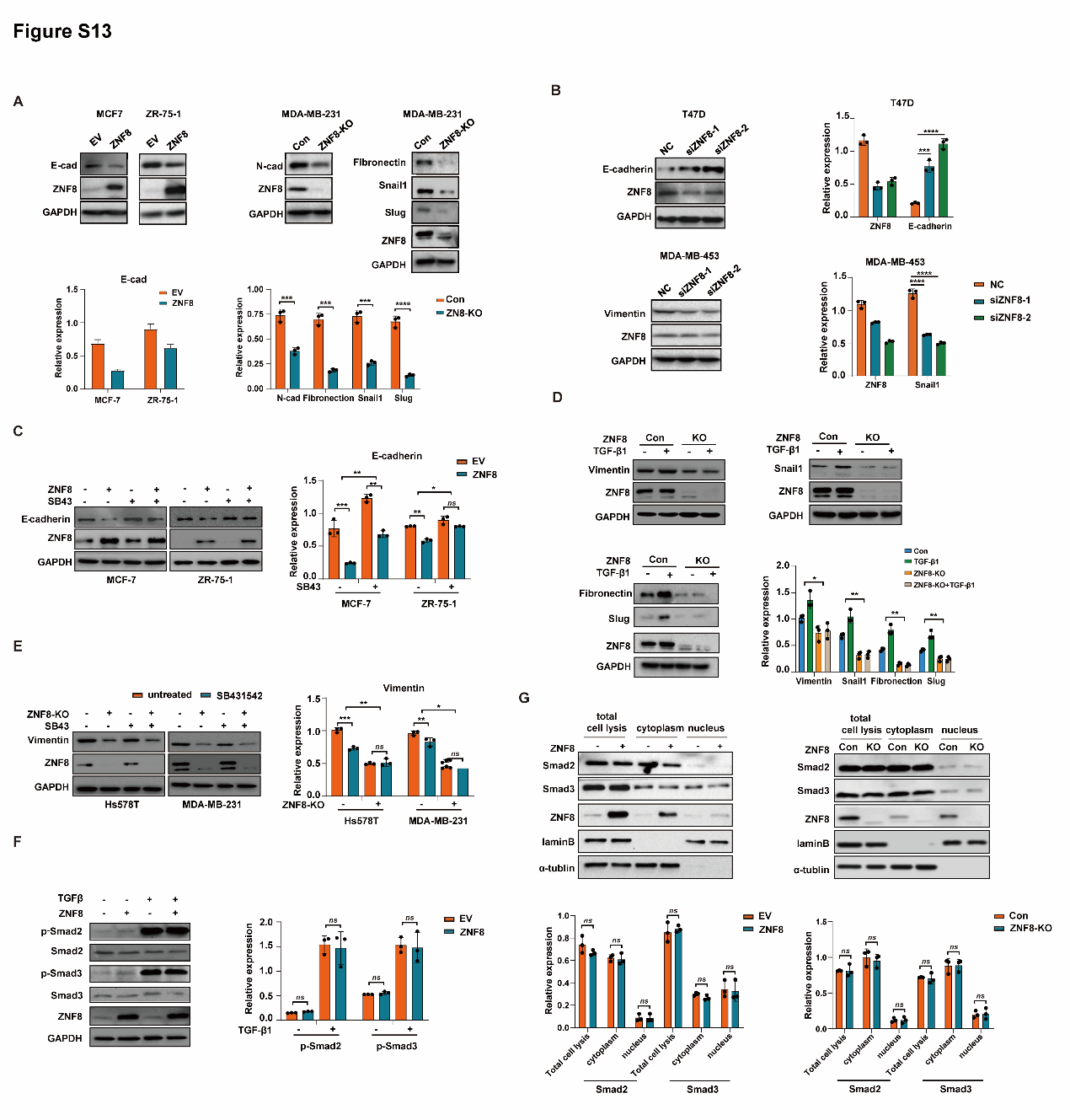


**Figure S13. The statistical analysis for the western blots**

Supplementary Tables

**Table S1**. Relationship between ZNF8 expression and the clinicopathologic characteristics in breast cancer patients Cohort 1

| Clinicopathologic characteristic | ZNF8 expression | | *p* |
| --- | --- | --- | --- |
|  | High Low | |  |
| **Age** |  |  | 0.056 |
| ≤50 | 61 | 41 |  |
| ＞50 | 21 | 29 |  |
| **Tumor size** |  |  | 0.327 |
| ≤2cm | 42 | 42 |  |
| ＞2cm | 40 | 28 |  |
| **Histological grade** |  |  | 0.007 |
| I | 20 | 34 |  |
| II | 38 | 20 |  |
| III | 24 | 16 |  |
| **Lymph nodes metastasis** |  |  | 0.035 |
| negative | 37 | 44 |  |
| positive | 45 | 26 |  |
| **ER status** |  |  | 0.157 |
| negative | 8 | 13 |  |
| positive | 74 | 57 |  |
| **PR status** |  |  | 0.855 |
| negative | 23 | 18 |  |
| positive | 59 | 52 |  |
| **HER2 status** |  |  | 0.027 |
| negative | 64 | 64 |  |
| positive | 18 | 6 |  |
| **Subtype** |  |  | 0.282 |
| Luminal A | 49 | 42 |  |
| Luminal B | 18 | 13 |  |
| HER2 | 11 | 6 |  |
| Triple Negative | 4 | 9 |  |

**Table S2. The clinical information of breast cancer patients Cohort 2**

| **Clinico-**  **pathologic characteristic** | **no metastasis (44)** | **Lung metastasis**  **(20)** | **Bone metastasis**  **(30)** | **Liver metastasis**  **(12)** | **Brain metastasis**  **(2)** |
| --- | --- | --- | --- | --- | --- |
| **Age** |  |  |  |  |  |
| ≤50 | 22 | 9 | 13 | 5 | 0 |
| ＞50 | 22 | 11 | 17 | 7 | 2 |
| **Tumor size** |  |  |  |  |  |
| ≤2cm | 11 | 3 | 10 | 3 | 0 |
| ＞2cm | 33 | 17 | 20 | 9 | 2 |
| **Menopausal status** |  |  |  |  |  |
| Yes | 21 | 12 | 17 | 8 | 1 |
| No | 23 | 8 | 13 | 4 | 1 |
| **Histological grade** |  |  |  |  |  |
| II | 29 | 12 | 22 | 10 | 2 |
| III | 13 | 8 | 18 | 2 | 0 |
| **Lymph nodes**  **metastasis** |  |  |  |  |  |
| negative | 9 | 8 | 3 | 3 | 1 |
| positive | 35 | 12 | 27 | 9 | 1 |
| **ER status** |  |  |  |  |  |
| negative | 13 | 10 | 8 | 6 | 1 |
| positive | 31 | 10 | 22 | 6 | 1 |
| **PR status** |  |  |  |  |  |
| negative | 13 | 10 | 11 | 5 | 1 |
| positive | 31 | 10 | 19 | 7 | 1 |
| **HER2 status** |  |  |  |  |  |
| negative | 37 | 9 | 25 | 9 | 0 |
| positive | 7 | 11 | 5 | 3 | 2 |

**Table S3. List of antibodies and regents used in the study.**

| **Antibody** | **Source** | **Catalog Number** | **Dilution/**  **Concentration** |
| --- | --- | --- | --- |
| Rabbit anti-ZNF8 | Thermo Fisher Scientific | PA5-57062 | 1:750(WB), 1:100(IP),1:100(IHC) |
| Rabbit anti-Smad2 | Cell Signaling Technology | 5339S | 1:1000(WB) |
| Rabbit anti-Smad3 | Cell Signaling Technology | 9523S | 1:1000(WB),1:100(IP),  1:50 (ChIP), 1:100(IHC) |
| Rabbit anti-P-Smad2 | Cell Signaling Technology | 18338T | 1:200(WB) |
| Rabbit  anti-P-Smad3 | Cell Signaling Technology | 9520T | 1:500(WB) |
| Rabbit anti- Smad2/3 | Cell Signaling Technology | 5678S | 1:100(IF) |
| Mouse anti-Smad4 | Santa Cruze | sc-7966 | 1:1000(WB),1:100(IF) |
| Mouse anti-E-cadherin | Cell Signaling Technology | 14472S | 1:500(WB) |
| Rabbit anti- N-cadherin | Cell Signaling Technology | 13116S | 1:500(WB) |
| Rabbit anti-Snail1 | Abcam | ab216347 | 1:1000(WB) |
| Mouse anti-Vimentin | Santa Cruze | sc-6260 | 1:500(WB) |
| Rabbit anti-Fibronectin | Proteintech | 15613-1-AP | 1:1000(WB) |
| Rabbit anti-Slug | Proteintech | 12129-1-AP | 1:1000(WB) |
| Mouse anti-GAPDH | Proteintech | 6004-1 | 1:1000(WB) |
| Mouse anti-Tubulin | Santa Cruze | 6A204 | 1:500(WB) |
| Rabbit anti-LaminB | Proteintech | 12987-1-AP | 1:1000(WB) |
| Flag-HRP | Sigma-Aldrich | A8592 | 1:1000(WB) |
| Myc-HRP | Sigma-Aldrich | Sc-40 | 1:500(WB) |
| Rabbit anti-Ly6G | Cell Signaling Technology | 87048T | 1:400 (IF) |
| Rabbit anti-Ki-67 | Abcam | ab15580 | 1:500(IHC) |
| PE Rat Anti-Mouse Ly6G | BD Biosciences | 551461 | 1:200 (FC) |
| Alexa Fluor® 700 Rat anti-CD11b | BD Biosciences | 557960 | 1:200 (FC) |
| FITC Rat Anti-Mouse CD45 | BD Biosciences | 553079 | 1:200 (FC) |
| Anti-Mouse CD16/CD32 | Tonbo | 70-0161-M001 | 1:200 (FC) |
| CY7- Hamster Anti-Mouse CD3e | BD Biosciences | 563024 | 1:200 (FC) |
| PE Rat Anti-Mouse F4/80 | BD Biosciences | 565410 | 1:200 (FC) |
| PE-Cy™7 Hamster Anti-Mouse CD11c | BD Biosciences | 558079 | 1:200 (FC) |
| Horizon™ BV711 Rat Anti-Mouse I-A/I-E | BD Biosciences | 563414 | 1:200 (FC) |
| PE/Fire(TM) 640 anti-mouse CD19 | BioLegend | 115574 | 1:200 (FC) |
| Rabbit anti-SMYD3 | Abcam | Ab228015 | 1:1000(WB), 1:100 (ChIP)  1:100 (IP), 1:100(IHC) |
| Rabbit anti-H3K4me3 | Abcam | ab8580 | 2 µg for 25 µg of chromatin( ChIP) |
| Rabbit anti-H3K27ac | Abcam | ab4729 | 2 µg for 25 µg of chromatin( ChIP) |
| Rabbit anti-H3k16ac | Thermo Fisher Scientific | MA5-27794 | 1:500(ChIP) |
| Rabbit anti-H3K36me2 | Abcam | ab176921 | 2 µg for 25 µg of chromatin( ChIP) |
| Rabbit anti-H3k27me3 | Abcam | ab192985 | 2 µg for 25 µg of chromatin( ChIP) |
| Normal Rabbit IgG | Cell Signaling Technology | ab172730 | 2 µg for 25 µg of chromatin( ChIP) |
| Alexa Fluor 594 Goat anti-Rabbit secondary antibody | Invitrogen | A-11012 | 1:1000(IF) |
| Alexa Fluor 488 Goat anti-Mouse secondary antibody | Invitrogen | A-11001 | 1:2000(IF) |
| Alexa Fluo488 Goat anti-Rabbit IgG (H+L) Cross-Adsorbed secondary antibody | Invitrogen | A-11008 | 1:500(IF) |
| PPD520/570/650 | PANOVUE | PPD650100100 | 1:200 |
| DAPI | Thermo Fisher Scientific | 62248 | 1:1000(IF) |
| SB431542 | Sellcek | S1067 | 5uM |
| TGF-β1 | PeproTech | 100-21C | 5ng/mL |
| BCI121 | MCE | HY-21972 | 20uM |
| Anti-mouse Ly6G-InVivo | Sellcek | A2158 | 200ug |
| Rat IgG2a isotype control-InVivo | Sellcek | A2123 | 200ug |

**Table S4. RT-qPCR/ Chip-qPCR primers details**

| **Human Gene** | **Forward（5’-3’）** | **Reverse（5’-3’）** |
| --- | --- | --- |
| ZNF8 | GGTAGCGGGAGTGATGTCTG | CTTCGGAAGCTCAGGACCTAT |
| MMP1 | GGGGCTTTGATGTACCCTAGC | TGTCACACGCTTTTGGGGTTT |
| SNAI1 | AGGATCTCCAGGCTCGAAAG | TCGGATGTGCATCTTGAGG |
| CCL5 | CCTGCTGCTTTGCCTACATTGC | ACACACTTGGCGGTTCTTTCGG |
| ANGPTL4 | GTCCACCGACCTCCCGTTA | CCTCATGGTCTAGGTGCTTGT |
| CXCL1 | AACCGAAGTCATAGCCACAC | TTGGATTTGTCACTGTTCAGC |
| CLDN2 | TTCATCGGCAACAGCATCG | GGTTATAGAAGTCCCGGATGA |
| CD44 | CTGCCGCTTTGCAGGTGTA | CATTGTGGGCAAGGTGCTATT |
| PDK1 | GGATTGCCCATATCACGTCTTT | TCCCGTAACCCTCTAGGGAATA |
| FGF5 | CACTGATAGGAACCCTAGAGGC | CAGATGGAAACCGATGCCC |
| GLI2 | CATGGAGCACTACCTCCGTTC | CGAGGGTCATCTGGTGGTAAT |
| PTHLH | ATTTACGGCGACGATTCTTCC | GCTTGGAGTTAGGGGACACC |
| Smad3 | GCGTGCGGCTCTACTACATC | GCACATTCGGGTCAACTGGTA |
| SETD7 | GGCCAGGGAGTTTACACTTAC | CTCATCAGGGTACACATAGGCTA |
| MLL1 | AAAGCCCTCGAAGGATTAAGC | AGCACTGACAACAGGCATGAT |
| MLL3 | TGGGTTCACCTAGAGTGTGAC | CTGGCTGTAAACGATCCATCTC |
| GAPDH | GGAGCGAGATCCCTCCAAAAT | GGCTGTTGTCATACTTCTCATGG |
| MMP1 promotor | AGTCAGATGTTCAAATGACTAAAGAGTT | AGTCACTACAGCACTTCACCCTTTAT |
| SNAI1 promotor | GAAATGAGACCACAGTAGGATCAAG | GGGGTTGATAAGTGACAGAAGGTA |
| CCL5 promotor | ATTGCCATTGAATCTCATTCCA | TGGAATGAGATTCAATGGCAAT |
| ANGPTL4 promotor | GTAGGGGAAAGGGGAGATGCCTG | CAGCCCAGCCGGAAAAGTAGG |
| CXCL1 promotor | CTCTGGTGCCAGAGGATATTC | AACAGCCAGAACCTGTTGCTG |
| CLDN2 promoter | TGCTAGCCAGGGACACCTAA | ACCTCTGTGAACCCAGGAGA |
| CD44  promoter | CAGAAAGGGGTCCTGGGTTC | CTGGTTCAAGCTTCCACAGC |
| PDK1 promoter | AAACCAGCTGGGCCGC | CACTGCACGGAATCCAAACG |
| FGF5  promoter | TGGAAATCGTGTCCTGGGG | GCGATAGAAACGGAGGGTTGA |
| GLI2  promoter | AGAGGCTAGTAGGCGCGA | AGAAGAGTGGTGCTCTCCCC |
| PTHLH promoter | CCACTTGTAGCGAAACCCAC | ATCGACGACACACGCACTT |

**Table S5. siRNA /sgRNA sequences information**

| **Name** | **Sequence（5’-3’）** | **Company** | |
| --- | --- | --- | --- |
| sgZNF8 | CACCGCGTCACGGTAGAGGATCCTC | BGI.tech |  |
| control sgRNA | UUCUCCGAACGUGUCACGUTT | BGI.tech |  |
| Negative control | UUCUCCGAACGUGUCACGUTT | GenePharma |  |
| siZNF8-1 | AGUAAUUUAUGGUAGAGUATT | GenePharma |  |
| siZNF8-2 | ACAUAGUGUACUCAUGGAATT | GenePharma |  |
| siSmad3-1 | GGAGAAAUGGUGCGAGAAG | GenePharma |  |
| siSmad3-2 | ACCUAUCCCCGAAUCCGAU | GenePharma |  |
| si-SETD7-1 | GGGCACCTGGATGACGGA | GenePharma |  |
| si-SETD7-2 | GGAGTGTGCTGGATATATT | GenePharma |  |
| siMLL1-1 | CCGGGCACTGTTAAACATTCCAC | GenePharma |  |
| siMLL1-2 | CCGGCGCCTAAAGCAGCTCTCAT | GenePharma |  |
| siMLL3-1 | CCAGAGAGCCAGAAAGAAA | GenePharma |  |
| siMLL3-2 | GCAAUUACUCUGCAUCCUA | GenePharma |  |

**Table S6. Plasmid primers details**

| **Plasmid Gene** | **Forward（5’-3’）** | **Reverse（5’-3’）** |
| --- | --- | --- |
| PGEX-4T-2-ZNF8 | ATTGAATTCTTATGGACCCCGAGGACGAA | AATGCGGCCGCCTATGTGGATTCTCTGAT |
| PLVX-3Flag-mZNF8 | CCCTCGAGATGGACCACCAGGACAAAGC | CCGGGATCCCTATCTAGATTCTCTGGTGTCAAACAGC |
| PET-28a-Smad3 | ATTGAATTCATGTCGTCCATCCTGCCT | ATAGCGGCCGCCTAAGACACACTGGAACA |
| pLV-Neo-Smad3 | TATCTCGAG ATGTCGTCCATCCTGCCT | ATAGCGGCCGCCTAAGACACACTGGAACA |
| pLV-Neo-Smad3-MH1 | ATAGAATTCGAATGTCGTCCATCCTGCCTTT | ATGCGGCCGCCTATGTCTCTACTCTCTGGTA |
| pLV-Neo-Smad3-linker | ATAGAATTCGACCAGTTCTACCTCCTGTGTT | AATGCGGCCGCCTATGGCTGCAGGTCCAAGT |
| pLV-Neo-Smad3-MH2 | ATAGAATTCGAGTTACCTACTGCGAGCCGGC | TATGCGGCCGCCTAAGACACACTGGAACAGC |
| pCMV-Myc-ZNF8- MH1 | ATTGAATTCTTGCCTGGGAGCCTCGATCTGA | ATAGCGGCCGCGGGTTTGTCCTGCACCT |
| pCMV-Myc-ZNF8-MH2 | GGCGAATTCTTTACAAATGTACTGACTGTGTGGG | ATACTCGAGCGCGTGCTTCCGCTGGT |
| pCMV-Myc-ZNF8-MH3 | ATTGAATTCTTTACAAATGTACTGACTGTGGGA | ATAGCGGCCGCGTGAGTTATCTGGTGCCGGAT |
| pCMV-Myc-ZNF8-C | ATTGAATTCTTGGGGAGAAGCCCTTTGA | ATAGCGGCCGCCTATGTGGATTCTCTGAT |
| pCMV-Myc-ZNF8-c | ATTGAATTCTTACCAGAGAGGAGCAGCCC | ATAGCGGCCGCCTATGTGGATTCTCTGATGTCAA |
| pCMV-Myc-ZNF8-△ZF | ATA GAATTCTTatggaccccgagg a | gcactcaaagggcttctccccgggtttgtcctgcacctggct |
| pCMV-Myc-ZNF8-△C | ATAGAATTCTTatggaccccgagg a | ATACTCGAGCGCGTGCTTCCGCTGGT |
| pLV-Neo-SMYD3 | ATTACTAGTATGGAGCCGCTGAAGGT | ATTGCGGCCGCTTAGGATGCTCTGATGTT |
| pLV-Neo-SMYD3-N | ATTACTAGTATGGAGCCGCTGAAGGTGGAA | ATTGCGGCCGCGCAGCTTTTAAGGCATTTGCATT |
| pLV-Neo-SMYD3-M | ATTACTAGTCCCAGATATCCTCCAGACT | ATTGCGGCCGCGTCCTGGGTTTGGCAA |
| pLV-Neo-SMYD3-C | ATTACTAGTAAGGATGCTGATATGCTAAC | ATTGCGGCCGCTTAGGATGCTCTGATGTT |
